# Galectin‐1 ameliorates perioperative neurocognitive disorders in aged mice

**DOI:** 10.1111/cns.13645

**Published:** 2021-05-04

**Authors:** Zhiwen Shen, Hui Xu, Wen Song, Chuwen Hu, Mingyan Guo, Jinfeng Li, Junhua Li

**Affiliations:** ^1^ Department of Anesthesiology Sun Yat‐sen Memorial Hospital Sun Yat‐sen University Guangzhou China; ^2^ Guangdong Provincial Key Laboratory of Malignant Tumor Epigenetics and Gene Regulation Sun Yat‐sen Memorial Hospital Sun Yat‐sen University Guangzhou China; ^3^ Department of Anesthesiology The Second Affiliated Hospital of Guangzhou University of Chinese Medicine Guangzhou China; ^4^ Guangdong Province Key Laboratory of Brain Function and Disease Zhongshan School of Medicine Sun Yat‐sen University Guangzhou China

**Keywords:** galectin‐1, IRAK1, microglia activation, neuroinflammation, perioperative neurocognitive disorders

## Abstract

**Introduction:**

The incidence of perioperative neurocognitive disorders (PND) is higher in the elderly patients undergoing surgery. Microglia activation‐mediated neuroinflammation is one of the hallmarks of PND. Galectin‐1 has been identified as a pivotal modulator in the central nervous system (CNS), while the role of galectin‐1 in PND induced by microglia‐mediated neuroinflammation is still undetermined.

**Methods:**

An exploratory laparotomy model anesthetized with isoflurane was employed to investigate the role of galectin‐1 on PND in aged mice. Open field test and Morris water maze were used to test the cognitive function 3‐ or 7‐days post‐surgery. The activation of microglia in the hippocampus of aged mice was tested by immunohistochemistry. Western blot, enzyme‐linked immunosorbent assay (ELISA), and quantitative real‐time polymerase chain reaction (qRT‐PCR) were employed to elucidate the underlying mechanisms.

**Results:**

Galectin‐1 attenuated the cognitive dysfunction induced by surgery in aged mice and inhibited microglial activity. Moreover, galectin‐1 decreased the expression level of inflammatory proteins (interleukin‐1β, interleukin‐6, and tumor necrosis factor‐α), and prevented neuronal loss in the hippocampus. Galectin‐1 inhibited the inflammation of BV2 microglial cells induced by lipopolysaccharide via decreasing the translocation of NF‐κB p65 and c‐Jun, while this kind of inhibition was rescued when overexpressing IRAK1.

**Conclusion:**

Our findings provide evidence that galectin‐1 may inhibit IRAK1 expression, thus suppressing inflammatory response, inhibiting neuroinflammation, and improving ensuing cognitive dysfunction. Collectively, these findings unveil that galectin‐1 may elicit protective effects on surgery‐induced neuroinflammation and neurocognitive disorders.

## Graphical abstract

Galectin‐1 ameliorated hippocampus‐dependent cognitive dysfunction induced by surgery. Furthermore, galectin‐1 mitigated surgery‐induce systemic inflammation and neuroinflammation induced by surgery through attenuating the transcriptional activity and translocation of c‐Jun and NF‐κB p65.

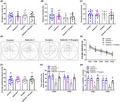

## 1 INTRODUCTION

Perioperative neurocognitive disorders (PND) are common postoperative complications, characterized by cognitive dysfunctions in the aged patient undergoing surgery and anesthesia. The occurrence of PND not only affects the quality of life and increases the burden of medical treatment, but also leads to increasing morbidity and mortality.^1^, ^2^, ^3^ PND are accompanied by obvious neuroinflammation characterized by microgliosis, reactive astrocytosis, and increased levels of pro‐inflammatory cytokines.^4^, ^5^ Despite multiple pathological events have been proposed to be associated with PND,^6^, ^7^ no effective clinical therapeutics treating PND has been developed.

As resident inflammatory cells in the central nervous system (CNS), microglia play a crucial role in neuroinflammation. Depending upon the nature of the original stimulation, the activated‐microglia exert multifarious role that could be either detrimental or beneficial. Microglia usually stay at a resting condition, but when exposed to exogenous antigens such as viruses or bacteria, microglia are rapidly activated and become neurotoxic.^8^ In addition, the activation of microglial may augment the transcriptional activity of c‐Jun and NF‐κB p65 and accelerate their translocation from the cytoplasm to the nucleus, leading to the increased level of inflammatory cytokines. Furthermore, activating microglia may promote to generate inflammatory proteins, such as interleukin‐1β (IL‐1β), interleukin‐6 (IL‐6), and tumor necrosis factor‐α (TNF‐α), giving rise to neuroinflammation and the ensuing cognitive deficits.^9^, ^10^ Consequently, the inactivation of microglial may attenuate neurocognitive dysfunction through attenuating the translocation of c‐Jun and NF‐κB p65 and neuroinflammation.

As a β‐galactoside‐binding protein, galectin‐1 has various biological activities and acts as a critical regulator in neuroinflammation. It has been shown that the expression of galectin‐1 is altered in neurological disorders and is associated with neuroinflammation.^11^, ^12^ In vitro, galectin‐1 modulates the function of microglia by reducing the LPS‐stimulated pro‐inflammatory cytokines (nitric oxide and TNF‐α) and contributing to the increased secretion of anti‐inflammatory cytokines (TGF‐β and IL‐10).^13^ Moreover, both exogenous and endogenous galectin‐1 play crucial role in deactivating microglia and facilitating a phenotype of alternative activation via regulating p38‐MAPK, CREB, and NF‐κB signaling pathways.^13^ Whether galectin‐1 attenuates perioperative neurocognitive disorders and the underlying mechanism are still undetermined.

In the present study, we provided evidence that galectin‐1 treatment attenuated surgery‐induced microglia activation and disruption of neuronal integrity in aged mice. This issue indicates how galectin‐1 exerted protective effect on PND, and demonstrates that galectin‐1 may be therapeutic by inhibiting IRAK1‐mediated translocation of c‐Jun and NF‐κB p65 and inflammatory response, which may retard the development of neuroinflammation and the ensuing neurocognitive dysfunction effectively.

## 2 METHODS AND MATERIALS

### 2.1 Animals

C57BL/6 mice (male, 26–38 g, 18 months old) were purchased from Guangdong Medical Laboratory Animal Research Institute. All animals were housed in polypropylene cages and the relative humidity was maintained at 50 ± 10%. The room temperature was maintained at 22°C with a 12 h light‐dark cycle. All of the experiment in this study were performed according to the National Institutes of Health Guidelines for experimental animals and all experimental protocols and procedures were approved by the animal care committee at Sun Yat‐sen University. All animal data acquired in the current study follows the ARRIVE guidelines.^14^ 120 mice in total were enrolled in the present study and they were randomly allocated into experimental groups. At the end of the experiment, all experimental animals were sacrificed by dislocation of cervical vertebra.

### 2.2 Surgery

Anesthetized with 1.5% isoflurane, a modified abdominal exploratory laparotomy was performed.^15^ In an induction chamber with 1.5% isoflurane and oxygen at 2 L/min, mice were first anesthetized. And after the mouse was exposed to isoflurane for 30 min, the abdominal region was shaved and sterilized thoroughly using 0.5% iodine. Then, a 1 cm median abdominal incision was made, so that the peritoneal cavity was penetrated. Next, the intestine, musculature, and viscera were explored. Using sterile 4‐0 chromic gut sutures, the peritoneal lining and skin were sutured. The entire surgical procedure was done under isoflurane anesthesia and lasted for 30 min. Neither surgery nor anesthesia was performed for the mice in the control group. During the process of surgery, the blood oxygen saturation of experimental mice was detected by a S_P_O_2_ probe for human infants.

### 2.3 Open field test (OFT)

The OFT was performed as we described before.^16^ In brief, OFT was performed in the center of open field device (50 cm × 50 cm × 37 cm), which consists of black floor and Plexiglass wall. By employing a video camera and the EthoVision™ XT 7.0 software (Noldus, Wageningen, Netherlands), the behaviors of experimental animals were recorded and analyzed. The chamber was cleaned thoroughly with 75% ethanol after each trial to avoid olfactory cues. The total traveling distance and speed were recorded and analyzed. All of the OFT were performed on POD 3.

### 2.4 Morris water maze (MWM)

The spatial memory performance of mice was assessed by MWM as described previously.^17^ Briefly, mice were first placed in the water maze pool to adapt to the environment for 2 days. During the training stage, mice were released from three different quadrants and trained to find the hidden platform for five consecutive days and the time taken (latency) to find the hidden platform was recorded. On the sixth day of MWM test, the hidden platform was removed, and mice were set free to swim at the starting point for 60 s to test their memory (target quadrant traveling time and platform‐crossing times were recorded). The MWM was performed on POD 3 and POD 7.

### 2.5 Experimental protocol

The mice were randomized into one of four experimental groups: (1) normal control group, (2) galectin‐1 group, (3) surgery group, and (4) galectin‐1+surgery group. The mice in the galectin‐1 and galectin‐1+surgery groups received an intraperitoneal injection of galectin‐1 (0.5 mg/kg, PeproTech, USA) diluted in 0.1 ml of sterile saline 30 min prior to the surgery. The dose (0.5 mg/kg) of galectin‐1 *in vivo* was chosen based on previous studies.^13^, ^18^ We harvested peripheral blood (by heart puncture) and hippocampus 6 h after surgery to assess the acute inflammatory response. Moreover, on POD 1 and POD 7, we also obtained hippocampus for analyses of immunofluorescence and Western blot.

### 2.6 Culture of primary microglial cell

Primary microglial cells were isolated from newborn mice (postnatal day 0–2). In brief, mice were euthanized and the brains were isolated in HBSS (H9269, Sigma), supplemented with 1% DNase (EN0521, Thermo) and 2.5% trypsin (15090–046, Gibco) for 15 min at 37°C. Cells were centrifuged at 1,000 × *g* for 5 min. The suspensions were incubated in the poly‐L‐lysine‐pretreated flasks at a density of 1 × 10^6^ and incubated until 90% confluent. The flask was shaken for 5 h at 180 rpm and centrifuged, so that we can collect the floating cells. Then, we suspended the pelleted cells and cultured them in DMEM/F12, which contained 1 mM of sodium pyruvate and 10% FBS in a 6‐cm dish precoated with poly l‐lysine (P8920, Sigma‐Aldrich) for 3 days to obtain the primary microglia.

### 2.7 BV2 cells

The murine microglial BV2 cell line was purchased from National Infrastructure of Cell Line Resource (China) and was cultured in Dulbecco's modified Eagle's medium (DMEM, Thermo Fisher, USA) supplemented with 10% fetal bovine serum (FBS), 0.1% penicillin/streptomycin at 37°C in a 5% CO2 humidified incubator. The BV2 cells were treated with LPS (100 ng/mL, Sigma, China) with or without galectin‐1 (5 μg/ml). The expression levels of inflammatory proteins and signaling molecules (c‐Jun and p65) in BV2 cells 24 h after LPS treatment were detected by ELISA and western blotting. Galectin‐1 was administered 30 min before LPS stimulation for all in vitro experiments.

### 2.8 Enzyme‐linked immunosorbent assay (ELISA)

The level of IL‐1β, IL‐6, and TNF‐α in peripheral blood, hippocampus, and cell supernatants were measured by ELISA kits (R&D Systems, MN) in strict accordance with manufacturer's instructions.

### 2.9 Quantitative real‐time PCR (qRT‐PCR)

Total RNA was extracted from cultured cells in accordance with the manufacturer's protocol (Invitrogen, USA). By employing Superscript First‐Strand cDNA Synthesis Kit (Invitrogen, USA), total RNA (1 μg) was reverse transcribed into cDNAs. SYBR Premix Ex Taq II kit (TAKARA, Japan) on LightCycler 480 System (Roche, Switzerland) was used to perform qRT‐PCR. The oligonucleotide sequences of the qRT‐PCR primers are listed in Table S1.

### 2.10 Plasmid and transfection

For overexpression of the IRAK1, BV2 cells were transfected with the plasmid pcDNA‐3.1‐IRAK1 (Hanheng Biotechnology, Shanghai, China) using Lipofectamine 2000 (Invitrogen, USA) according to the manufacturer's instructions. Plasmid pcDNA‐3.1 was used as a control. In brief, BV2 cells were seeded in DMEM (10% FBS) and cultured until they achieved 80% confluence. We then replaced the culture medium with low‐serum media (0.5% FBS), and then BV2 cells were transfected with 10 μg/well of plasmid. Cells were collected for further study at 48 h post‐transfection. The efficiency of IRAK1 overexpression was measured qRT‐PCR as indicated in Figure 8E.

### 2.11 Nitrite assay

Griess assay was employed to detect the accumulation of nitrite (NO_2_^−^) in cultured supernatant. Microglial cells were first planted into 96‐well plates at the density of 5 × 10^4^ cells/well, and then the cells were treated with LPS (100 ng/ml) for 24 h. Next, 50 μl Griess reagent and 50 μl culture supernatant fluids were mixed together for 15 min at 37°C, and the absorbance was determined at 540 nm.

### 2.12 Luciferase reporter assay

Luciferase reporter assay was conducted as previously described. In brief, by employing Lipofectamine 3000 reagent (Invitrogen), BV2 cells were co‐transfected with Renilla luciferase plasmid (pRL‐SV40‐C) and AP‐1 luciferase reporter plasmid or NF‐κB luciferase reporter plasmid for 48 h. Then, the BV2 cells were treated with LPS (100 ng/mL) for 24 h with or without galectin‐1 (5 μg/ml). Reporter activity was detected using Dual Luciferase Reporter Gene Assay Kit (Beyotime, China) according to the manufacturer's instructions.

### 2.13 MTT cell viability assay

MTT assay was used to measure the cell viability in strict accordance with the manufacturer's instruction. In brief, cells administered with LPS for 6 h with or without galectin‐1 were seeded at the density of 1 × 10^4^ cells/well in 96‐well plates and cultured for 24 h at 37°C. MTT (Sigma‐Aldrich, M2128) was added to the cells at a final concentration of 0.5 mg/ml, and the cells were incubated at 37℃ for another 4 h. Finally, cells were washed with PBS for three times and formazan crystals were dissolved in 100 ml DMSO. The absorbance was read at 490 nM using a microplate reader (PerkinElmer, USA).

### 2.14 Western blot

Proteins in cell culture media and hippocampal tissues were lysed in RIPA lysis buffer with protease inhibitor cocktail. Protein samples were subjected to 10% SDS‐PAGE and transferred to PVDF membranes. The membranes were subsequently incubated with antibodies iNOS (Abcam #ab178945, USA, 1:500), IL‐1β (Abcam #ab234437, 1:500), IL‐6 (Abcam #ab233706, 1:500), TNF‐α (Abcam #ab215188, USA, 1:500), c‐Jun (CST #9165, USA, 1:500), p65(CST #8242, USA, 1:500), IRAK1 (CST #4504, USA, 1:500), β‐actin (CST #3700, USA, 1:2000). Then the membranes were incubated with HRP‐conjugated secondary antibodies for 1 h at room temperature and detected with enhanced chemiluminescence. The results were analyzed by using ImageJ software (National Institutes of Health, USA).​ The original western blot images have been attached in the Appendix S1.

### 2.15 Immunofluorescence

The mice were sacrificed, and their brains were fixed in 4% paraformaldehyde at 4°C for 24 h and then embedded in paraffin. The brain tissues were cut into 10‐μm‐thick slices. The slices were blocked with 10% donkey serum for 1 h, incubated with anti‐Iba‐1 (Abcam, #ab178847, 1:200) or anti‐NeuN (CST #24307, USA, 1:200) antibodies overnight at 4°C. After washing with PBS solution for three times, sections were incubated with secondary antibodies at room temperature for 1 h. After being washed, the sections were incubated with DAPI for nuclear staining. By using ImageJ software (National Institutes of Health, USA), Iba‐1‐positive cells and NeuN‐positive cells were counted with a DAPI counterstain.

### 2.16 Statistical analysis

The sample size was calculated by using the SPSS 16.0 software (SPSS, Chicago, USA) to achieve an 80% power at a significance level of 0.05. Two‐way ANOVA was employed to evaluate latency in the MWM test and student's t test was used to assess the differences between two groups. Whether the data were normally distributed is detected by the Shapiro‐Wilk normality test. Data were expressed as means ±SD and analyzed by one‐way ANOVA followed by Tukey's post hoc test. The GraphPad Prism software (version 7.0, CA, USA) was employed to perform the statistical analyses. In all cases, statistical significance was accepted at *p* < 0.05.

## 3 RESULTS

### 3.1 Galectin‐1 ameliorated hippocampus‐dependent cognitive dysfunction induced by surgery

**FIGURE 1:**
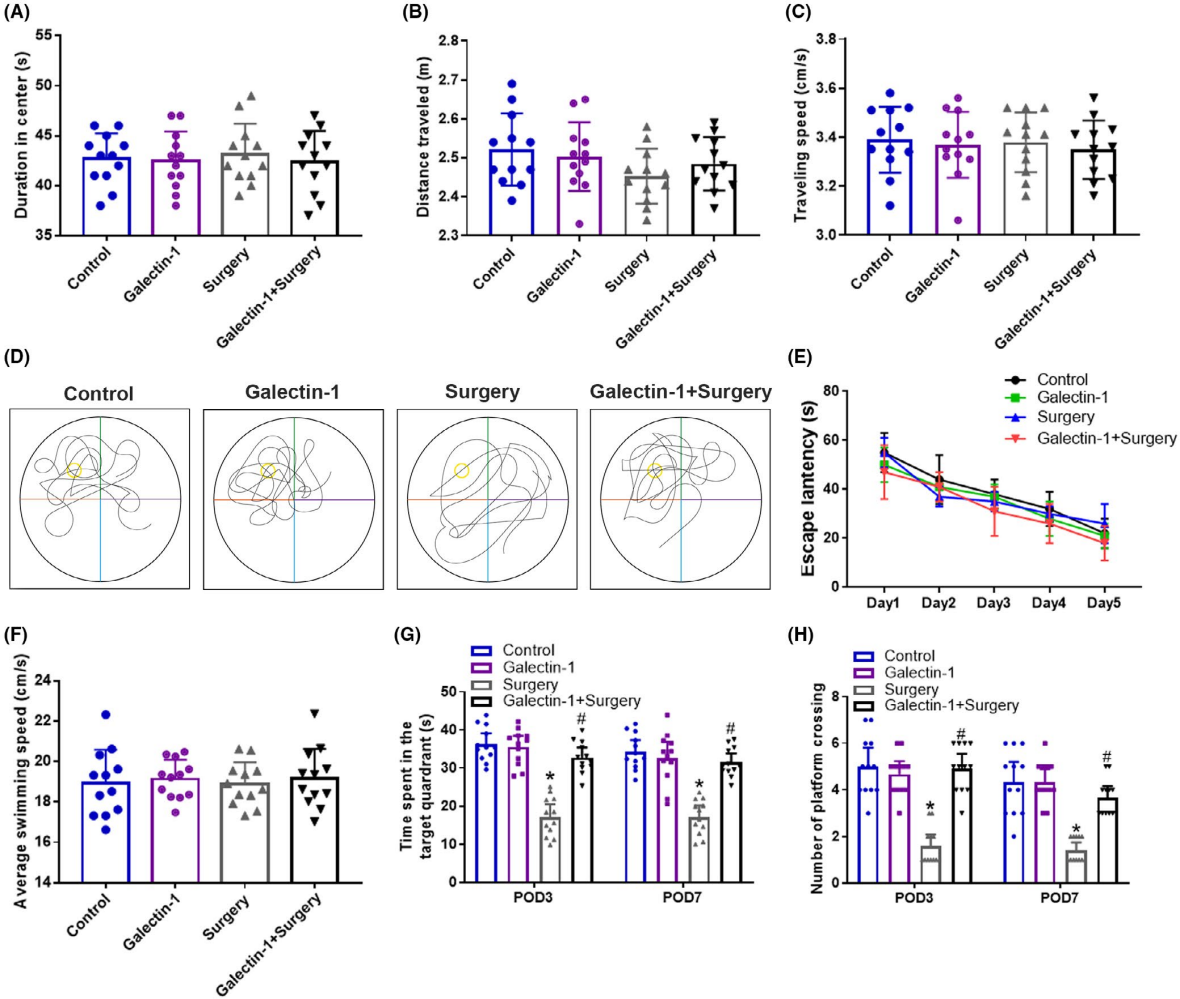
Galectin‐1 attenuated neurocognitive dysfunction induced by surgery (*n* = 12 in each group). Open field test was performed on POD 3. Duration in center (A), distance travelled (B), and travelling speed (C) in open field test. The representative swimming trace of the aged rats during Morris water maze testing with hidden platform (D). Escape latency (E) and average swimming speed (F) in the training phase of MWM with visible platform. The time spent in the target quadrant (G) and number of platform crossing (H) in the probe testing of MWM with hidden platform on POD 3 and POD 7. Data are presented as mean ± SD. *p < 0.05 versus the control and galectin‐1 groups, ^#^ *p* < 0.05 versus the surgery group

To investigate the physiological role of galectin‐1 on cognitive dysfunction induced by surgery, OFT was first performed to test the locomotor activity on postoperative day 3 (POD 3). As shown by the results of OFT, no significant change was observed in duration in center (Figure 1A, *p* > 0.05), distance traveled (Figure 1B, *p* > 0.05), and traveling speed (Figure 1C, *p* > 0.05) among the four groups, indicating that no spontaneous locomotor activity decline was caused by the surgery.

Then, on POD 3 and POD 7, the hippocampus‐dependent memory was evaluated by WWM (Figure 1D). As show by the results of training session in WWM, no remarkable difference was observed in escape latency (Figure 1E, *p* > 0.05) and average swimming speed (Figure 1F, *p* > 0.05) among the four groups, suggesting that the mice in the study have learned to find the hidden platform after training for five consecutive days. During the probe session, the time spent in the target quadrant (Figure 1G, *p* < 0.05) and the number of platform crossing (Figure 1H, *p* < 0.05) significantly decreased in surgery group on both POD 3 and POD 7, when compared to control group, indicating surgery had induced cognitive dysfunction. However, when pretreated with galectin‐1, the decline in cognitive dysfunction induced by surgery (Figure 1G,H, *p* < 0.05) on both POD 3 and POD 7 was rescued, demonstrating that galectin‐1 may elicit protective effect on the cognitive impairment induced by surgery.

### 3.2 Galectin‐1 mitigated systemic inflammation and neuroinflammation induced by surgery

**FIGURE 2:**
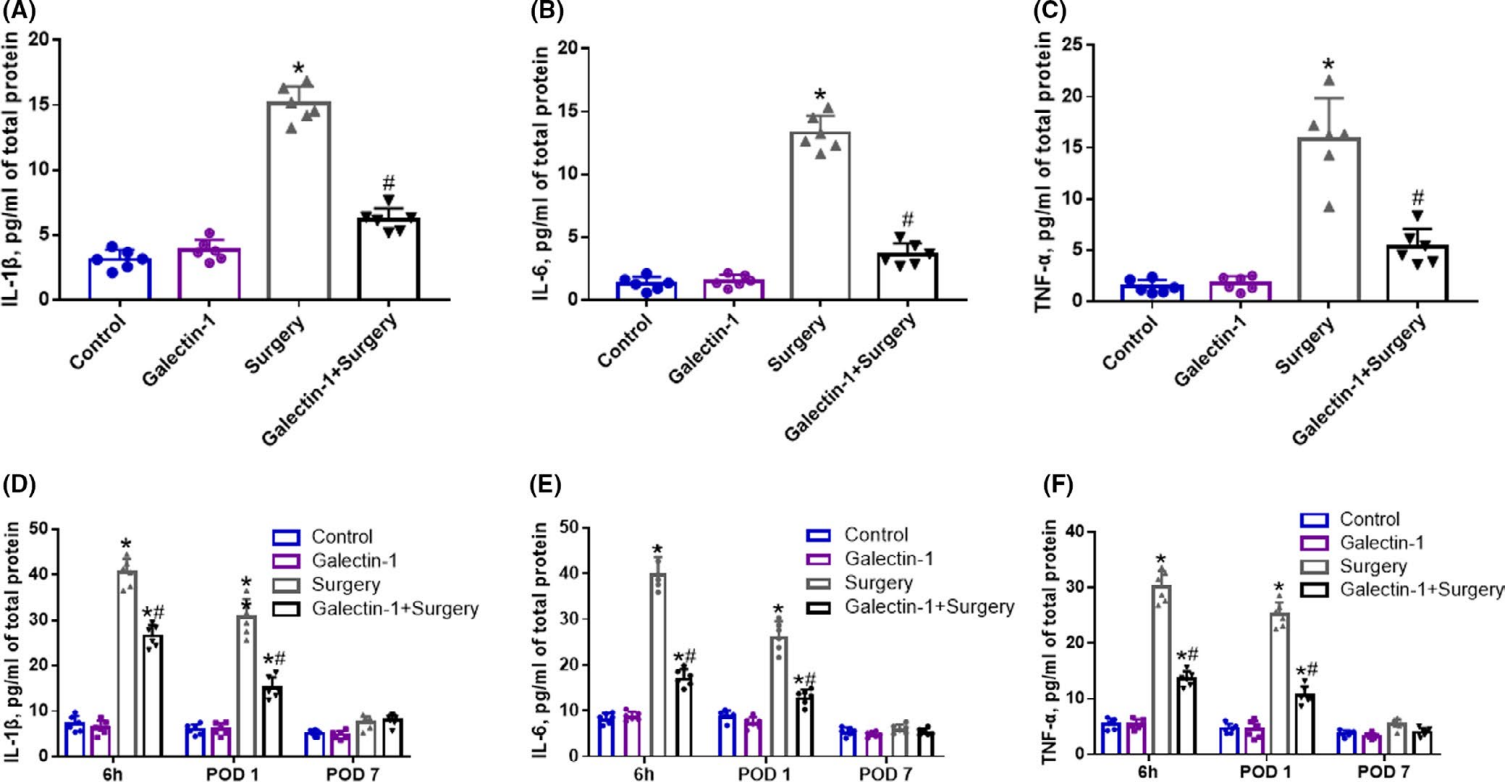
The effects of galectin‐1 on systemic inflammation and neuroinflammation induced by surgery (*n* = 6 in each group). The protein expression levels of IL‐1β (A), IL‐6 (B), and TNF‐α (C) in the peripheral blood harvested 6 h after surgery. The protein expression levels of IL‐1β (D), IL‐6 (E), and TNF‐α (F) at different time points in the hippocampus. Data are presented as the mean ±SD. *p < 0.05 versus the control and galectin‐1 groups, ^#^ *p* < 0.05 versus the surgery group

After surgery, we then collected the peripheral blood and the hippocampus sample to test the acute inflammatory response. In peripheral blood, the expression levels of IL‐1β, IL‐6, and TNF‐α increase significantly 6 h after surgery, while pretreated with galectin‐1 remarkably decreased the surgery‐induce elevation of these inflammatory proteins (Figure 2A–C, *p* < 0.05). In term of neuroinflammation in hippocampus, analogical results were observed: galectin‐1 significantly rescued the upregulated inflammatory factors (including IL‐1β, IL‐6, and TNF‐α) induced by surgery in the hippocampus 6 h after surgery (Figure 2D–F, *p* < 0.05). The expression levels of inflammatory cytokine in hippocampus remarkably elevated in the surgery group in comparison with the normal group. Nevertheless, on POD 1, pretreatment with galectin‐1 attenuated the expression levels of IL‐1β, IL‐6, and TNF‐α in hippocampus (Figure 2D–F, *p* < 0.05). On POD 7, no significant difference was observed in the expression levels of inflammatory cytokine among the four groups (Figure 2D–F, *p* > 0.05), indicating that the inflammation in the aged mice might have abated as time goes on. All of these results suggested that galectin‐1 might inhibit surgery‐induced systemic inflammation and neuroinflammation.

### 3.3 Pretreatment with galectin‐1 suppressed microglia activation in the hippocampus

**FIGURE 3:**
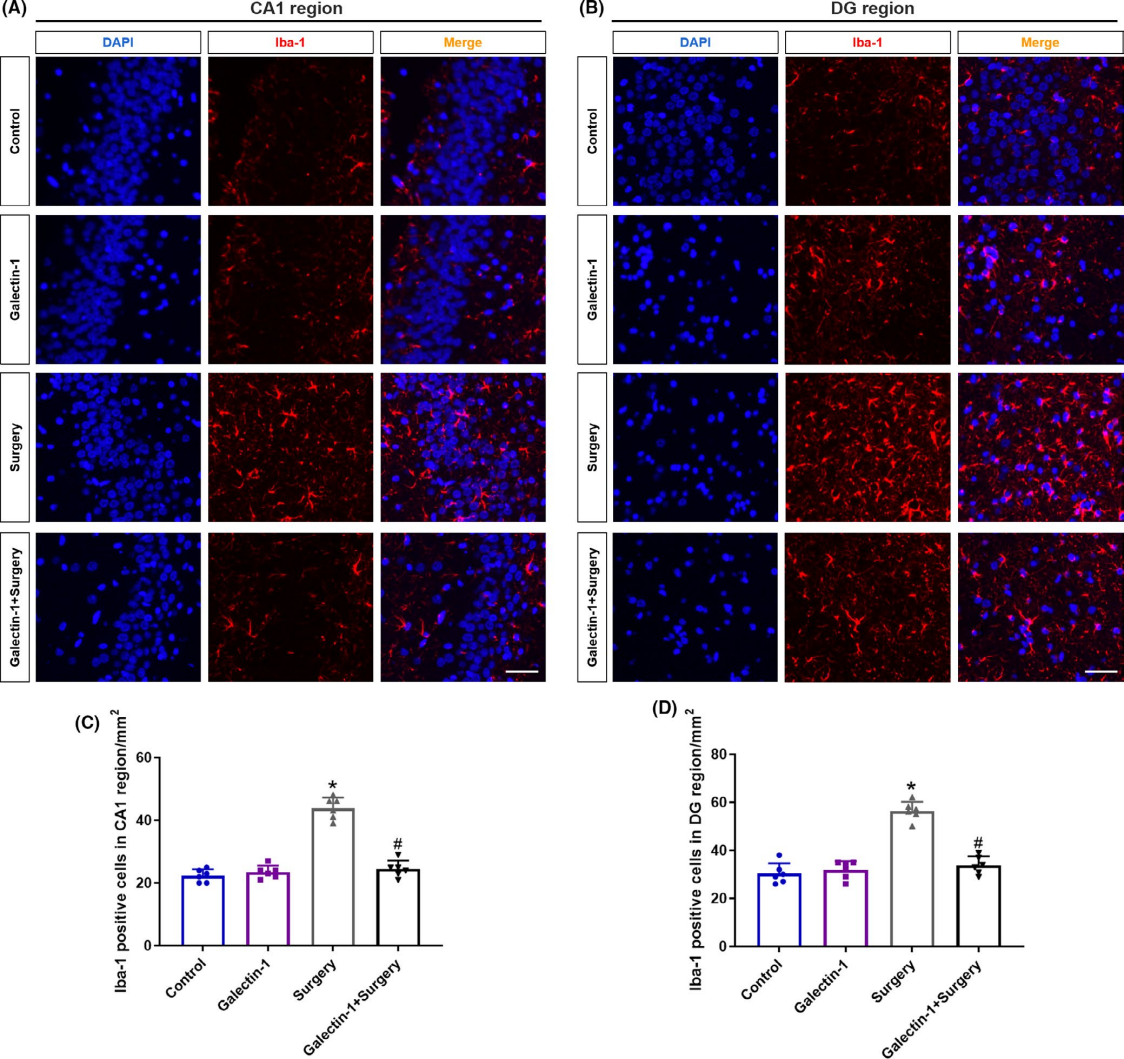
Galectin‐1 mitigated surgery‐induced microglial activation (*n* = 6 in each group). Representative images of Iba‐1 positive cells in CA1 (A) and DG regions (B). Red staining indicated activated microglia. Scale bars = 200 μm. Quantification of Iba1‐positive cells in CA1 region (C) and DG region (D). Data are presented as the mean ± SD. *p < 0.05 versus the control and galectin‐1 groups, ^#^ *p* < 0.05 versus the surgery group

Since microglia activation may promote neuroinflammation in the process of PND, we measured the microglial activation through immunofluorescence marked by ionized calcium‐binding adaptor molecule 1 (Iba‐1) on POD 7. The average number of Iba1‐positive cells per field in CA1 region (Figure 3A,C, *p* < 0.05) and DG region (Figure 3B,D, *p* < 0.05) was significantly increased after surgical trauma. However, pretreatment with galectin‐1 remarkably reversed these elevation (Figure 3A–D, *p* < 0.05), suggesting that galectin‐1 may suppress the activation of microglial and attenuate the ensuing inflammatory response.

### 3.4 Galectin‐1 protects neurons from inflammatory cytokines induced by surgery

**FIGURE 4:**
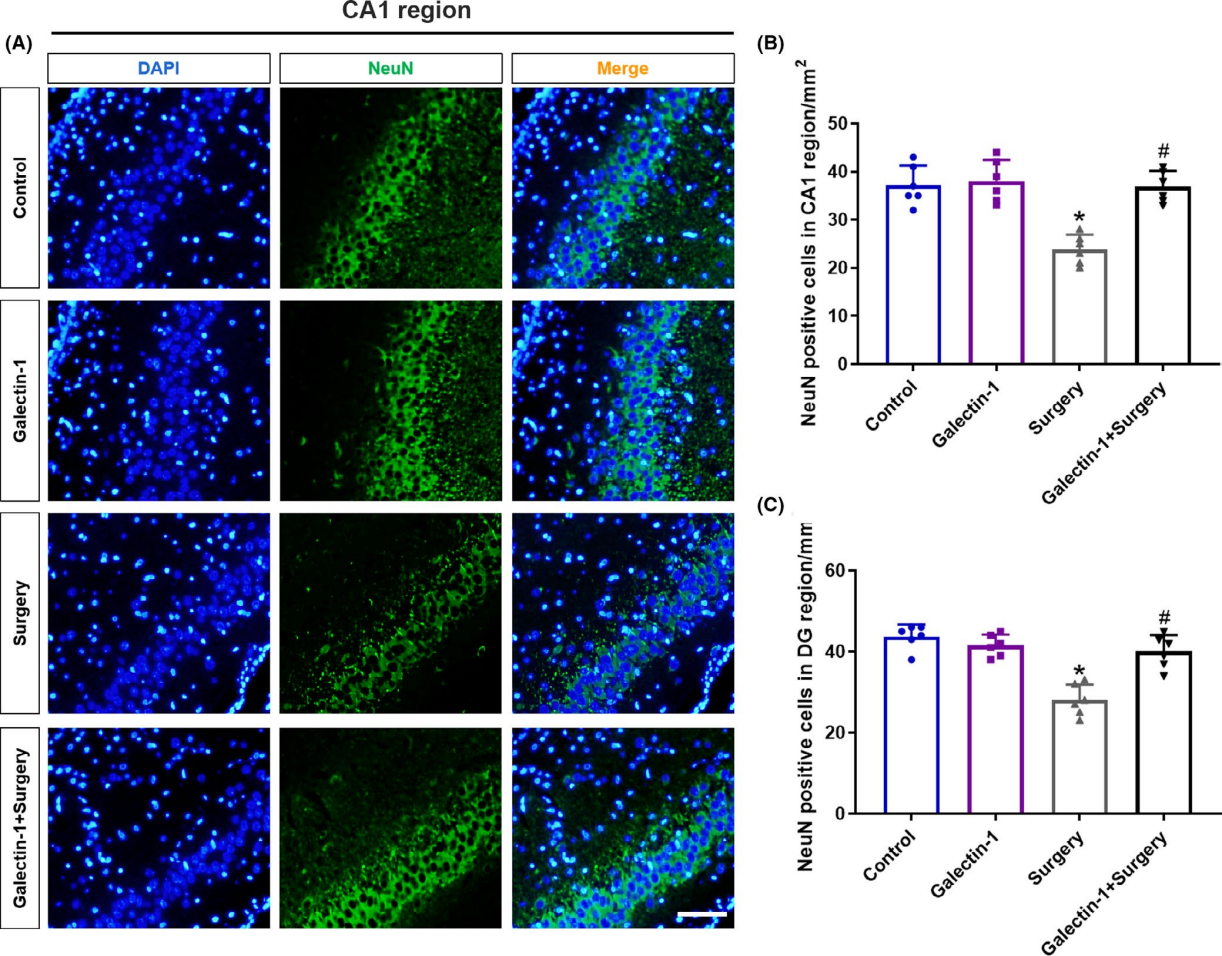
Galectin‐1 protects neurons from inflammatory cytokines induced by surgery (*n* = 6 in each group). Representative immunofluorescence images of NeuN‐positive cells in CA1 region (A). Scale bars =200 μm. Quantification of NeuN‐positive cells in CA1 region (B) and DG region (C). Data are presented as the mean ± SD. *p < 0.05 versus the control and galectin‐1 groups, ^#^ *p* < 0.05 versus the surgery group

Since neuroinflammation exerts detrimental effect on neurons, we then tested the neuronal integrity among the four groups using a NeuN antibody, which indicates the neuronal integrity. As shown by the results of immunofluorescence, the number of NeuN‐positive cells in CA1 region (Figure 4A,B, *p* < 0.05) and DG region (Figure 4C, *p* < 0.05) remarkably decreased after exposing to surgery, while pretreatment with galectin‐1 significantly rescued the reduction caused by surgery, compared with the control group in CA1 region and DG region. Therefore, we suggested that galectin‐1 protected neurons from surgery‐induced inflammatory cytokines in vivo.

### 3.5 Galectin‐1 reduced NO release induced by LPS through elevating the expression of iNOS

**FIGURE 5:**
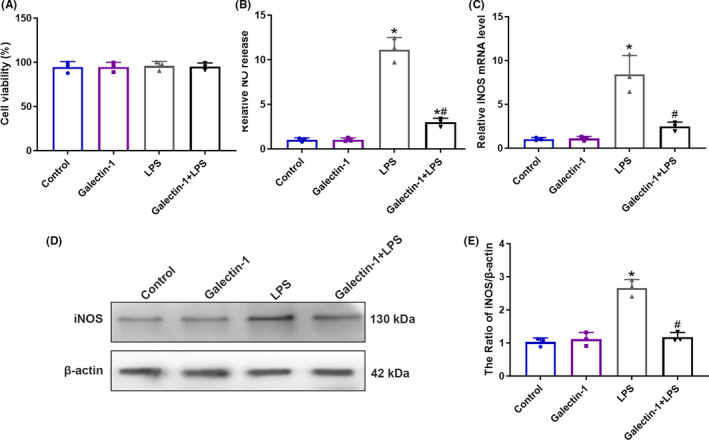
Galectin‐1 reduced NO release induced by LPS through elevating the expression of iNOS in BV2 cells (*n* = 3 in each group). Cell viability 6 h after different treatment detected by CCK8 assay (A). NO production in four groups detected by Griess assay (B). Relative iNOS mRNA level of BV2 cells 6 h after different treatment detected by qRT‐PCR (C). The representative western blot images (D) and quantification analysis (E) of iNOS expression of BV2 cells in four groups. Data are presented as the mean ± SD. *p < 0.05 versus the control and galectin‐1 groups, ^#^ *p* < 0.05 versus the LPS group

To further explore the protective effect of galectin‐1 in PND, we then examine the role of galectin‐1 in vitro. BV2 cell treated with LPS (100 ng/ml) was used as a neuroinflammation model, which is a common approach studying PND.^19^, ^20^ By employing nitrite assays, the effect of galectin‐1 on microglial activation was determined. There is no significant difference among the four groups on cell viability (Figure 5A, *p* > 0.05). Compared with the control group, the production of nitric oxide (NO) significantly increased in BV2 cells treated with LPS (Figure 5B, *p* < 0.05). However, galectin‐1 treatment reversed this elevation induced by LPS (Figure 5B, *p* < 0.05). Inducible nitric oxide synthase (iNOS), induced in microglia by inflammatory mediators such as LPS and cytokines, contribute to the release of NO.^21^ Consequently, we used iNOS production to evaluate whether galectin‐1 affects the release of NO. As shown by the results, galectin‐1 significantly decreased the mRNA (Figure 5C, *p* < 0.05) and protein (Figure 5D,E, *p* < 0.05) expression levels of iNOS, when compared with the LPS group, suggesting that galectin‐1 mitigated LPS‐induced NO release through reducing the expression of iNOS.

### 3.6 Galectin‐1 decreased LPS‐induced inflammatory cytokines production

**FIGURE 6:**
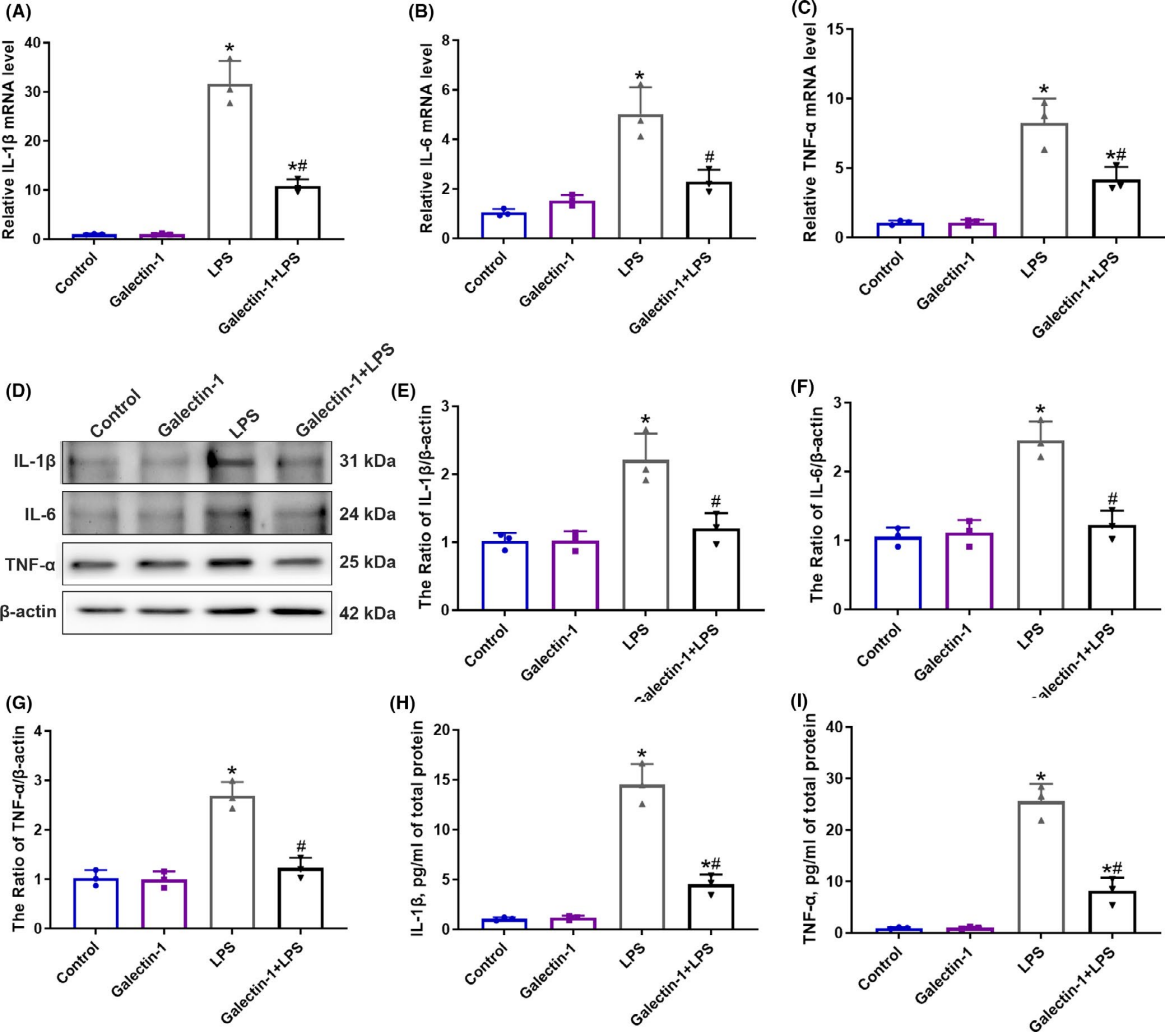
Galectin‐1 decreased LPS‐induced inflammatory cytokines production (*n* = 3 in each group). BV2 cells were treated with LPS (100 ng/ml) for 4 h. The relative mRNA level of IL‐1β (A), IL‐6 (B), and TNF‐α (C) tested by qRT‐PCR. The representative immunoblot images (D) and quantitative analysis of IL‐1β (E), IL‐6 (F), and TNF‐α (G) expression in BV2 cells. The relative secreted protein expression of cytokines IL‐1β (H) and TNF‐α (I) in the supernatant tested by ELISA assay. Data are presented as the mean ± SD. *p < 0.05 versus the control and galectin‐1 groups, ^#^ *p* < 0.05 versus the LPS group

We then assessed the effect of galectin‐1 on pro‐inflammatory cytokines to explore the influence of galectin‐1 on the inflammatory response. The expression levels of the pro‐inflammatory cytokines (IL‐1β, IL‐6, and TNF‐α) were measured in BV2 cells. When exposed to LPS, the mRNA production of IL‐1β, IL‐6, and TNF‐α show remarkable increase, but galectin‐1 administration significantly reduced the elevated mRNA levels of IL‐1β, IL‐6, and TNF‐α (Figure 6A–C, *p* < 0.05). Moreover, we tested the protein expression of IL‐1β, IL‐6, and TNF‐α 24 h after LPS treatment. When administered with LPS, the protein levels of IL‐1β, IL‐6, and TNF‐α remarkably elevated among the four groups, but galectin‐1 treatment reduced the LPS‐induced increased protein level of IL‐1β, IL‐6, and TNF‐α (Figure 6D–G, *p* < 0.05). In addition, the secreted protein levels of IL‐1β and TNF‐α were assayed 24 h after administration with LPS by ELISA assay, and the results are in accord with the results presented above. After LPS administration, the expression levels of secreted IL‐1β and TNF‐α remarkably increased, while galectin‐1 significantly decreased the elevation induced by LPS (Figure 6H,I, *p* < 0.05).

### 3.7 Galectin‐1 attenuated the transcriptional activity and translocation of c‐Jun and NF‐κB p65

**FIGURE 7:**
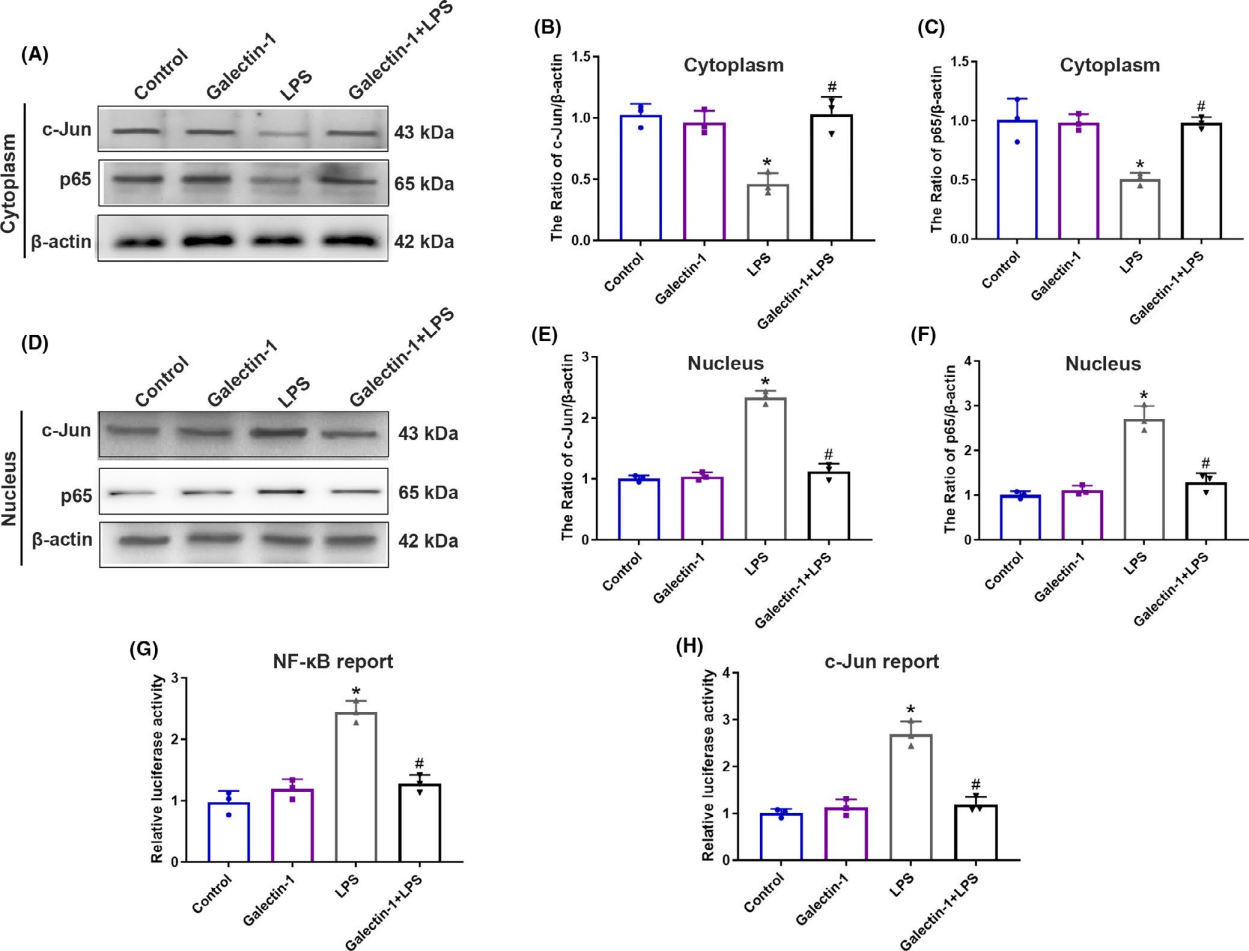
Galectin‐1 attenuated the transcriptional activity and translocation of c‐Jun and translocation (n = 3 in each group). Representative immunoblot images of c‐Jun and p65 expression in cytoplasm of BV2 cells (A). The quantitative analysis of c‐Jun (B) and p65 (C) in cytoplasm of BV2 cells. Representative immunoblot images of c‐Jun and p65 expression in nucleus of BV2 cells (D). The quantitative analysis of c‐Jun (E) and p65 (F) in nucleus of BV2 cells. BV2 cells were co‐transfected with Renilla luciferase plasmid (pRL‐SV40‐C) and AP‐1 luciferase reporter plasmid or NF‐κB luciferase reporter plasmid for 48 h, and NF‐κB (G) and c‐Jun (H) reporter activities were analyzed following LPS stimulation. Data are presented as the mean ±SD. *p < 0.05 versus the control and galectin‐1 groups, ^#^ *p* < 0.05 versus the LPS group

The proteins of the MAPK and NF‐κB pathways are the main modulator in pro‐inflammatory cytokine production and secretion.^22^, ^23^ According to the results of NO release and cytokine production, whether galectin‐1 affects cytokine production controlled by MAPK‐dependent AP‐1 transcription factors and NF‐κB p65 was explored. The nuclei of BV2 cells were separated from the cytoplasm after exposing to LPS for 30 min. Stimulated by LPS, the translocation of c‐Jun and NF‐κB p65 from the cytoplasm to the nucleus showed remarkable increase in comparison with the control group (Figure 7A–F, *p* < 0.05). However, pretreatment with galectin‐1 rescued the elevation induced by LPS (Figure 7A–F, *p* < 0.05). Furthermore, we then tested the transcriptional activity of c‐Jun and NF‐κB p65 in BV2 cells. Pretreatment with galectin‐1 significantly attenuated the increased transcriptional activity of c‐Jun (Figure 7G, *p* < 0.05) and NF‐κB p65 (Figure 7H, *p* < 0.05) following LPS stimulation. Our finding suggest that galectin‐1 decreases the release of NO and inflammatory cytokine by attenuating the transcriptional activity and translocation of c‐Jun and NF‐κB p65.

### 3.8 Overexpressing IRAK1 rescued the protective effect on the production of inflammatory factors exerted by galectin‐1

**FIGURE 8:**
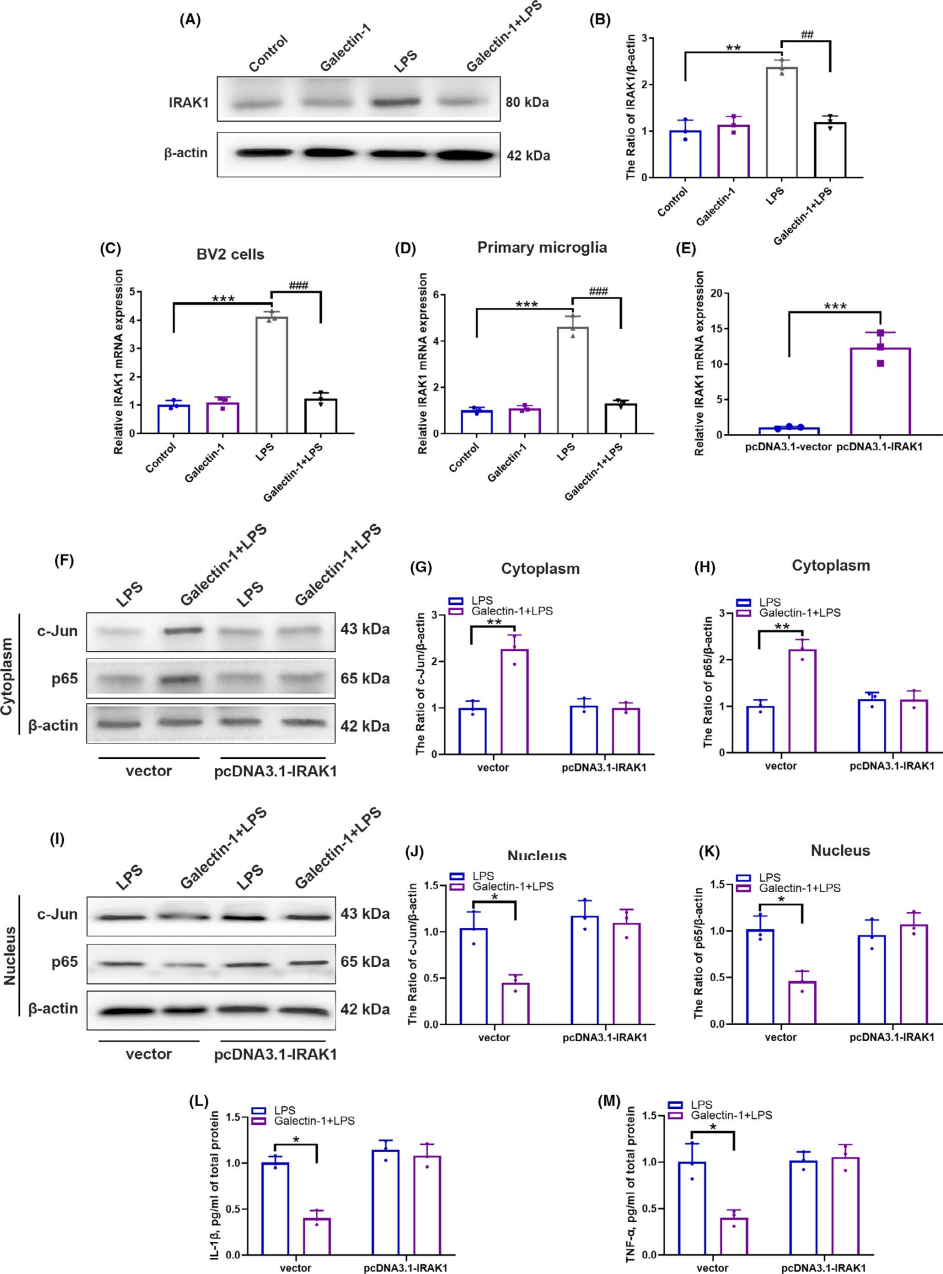
The protective effect of galectin‐1 attenuating inflammation depended on the inhibition of IRAK1 (n = 3 in each group). Representative immunoblot images of IRAK1 in BV2 cells (A). The quantitative analysis of IRAK1 in BV2 cells (B). The relative mRNA level of IRAK1 in BV2 cells (C) and primary microglia (D) tested by qRT‐PCR. After being transfected with pcDNA3.1‐IRAK1 for 48 h, BV2 cells were treated with galectin‐1 for 30 min, with or without subsequent LPS stimulation. Relative mRNA level of IRAK1 in BV2 cells transfected with pcDNA3.1‐vector or pcDNA3.1‐IRAK1 tested by qRT‐PCR (E). Representative immunoblot images of c‐Jun and p65 expression in cytoplasm of BV2 cells (F). The quantitative analysis of c‐Jun (G) and p65 (H) in cytoplasm of BV2 cells. Representative immunoblot images of c‐Jun and p65 expression in nucleus of BV2 cells (I). The quantitative analysis of c‐Jun (J) and p65 (K) in nucleus of BV2 cells. The relative secreted protein expression of cytokines IL‐1β (L) and TNF‐α (M) in the supernatant tested by ELISA assay. Data are presented as the mean ±SD. *p < 0.05, **p < 0.01, ***p < 0.001

We then explored the mechanism by which galectin‐1 attenuated the transcriptional activity and translocation of c‐Jun and NF‐κB p65. As shown by our results, the protein expression level of IRAK1 showed significant reduction in BV2 cells when treated with galectin‐1 (Figure 8A,B, *p* < 0.05). Moreover, the mRNA expression of IRAK1 in BV2 cells (Figure 8C, *p* < 0.05) and primary microglia (Figure 8D, *p* < 0.05) also showed significant reduction when pretreated with galectin‐1.

Next, pcDNA‐3.1‐IRAK1 (Figure 8E) were transfected in BV cells for 24 h to further determine whether galectin‐1 decreased cytokine production through IRAK1. We separated the nuclei from the cytoplasm after the BV2 cells were treated with LPS for 30 min. As indicated by our results, the translocation of c‐Jun and NF‐κB p65 from the cytoplasm to the nucleus significantly suppressed when pretreated with galectin‐1, while this kind of protective effect was rescued when transfected with pcDNA‐3.1‐IRAK1 (Figure 8F–K, *p* < 0.05). Then we detected whether overexpressing IRAK1 could rescue the galectin‐1‐mediated ameliorating effect on inflammatory factors production. After the BV2 cells were transfected with pcDNA‐3.1‐IRAK1 for 24 h followed by stimulation with LPS for 30 min, the supernatants were collected and analyzed by ELISA. The galectin‐1 induced decreased pro‐inflammatory cytokines of IL‐1β (Figure 8L, *p* < 0.05) and TNF‐α (Figure 8M, *p* < 0.05) was rescued when overexpressing IRAK1. These results indicated that galectin‐1 might attenuate the inflammatory response via inhibiting IRAK1 expression.

## 4 DISCUSSION

Emerging emphasis has been put on the role of neuroinflammation in the progression of PND.^24^, ^25^ Characterized by producing inflammatory mediators that facilitate the development and severity of PND, microglia play a crucial role in the progression of PND.^26^, ^27^ In a mouse model of PND, we investigate the correlation between galectin‐1 treatment and neurocognitive dysfunction. Our findings suggest that treatment with galectin‐1 attenuates the surgery‐induced neuroinflammation and hippocampus‐dependent cognitive dysfunction. By suppressing the activation of microglial effectively, galectin‐1 decreased the translocation of c‐Jun and NF‐κB p65, inhibited the inflammatory response, and thus ameliorating the ensuing neurocognitive dysfunction. This protective effect of galectin‐1 seemed to depend on inhibiting IRAK1 expression.

PND induced by surgery are relatively common, which occurs in 10%‐54% of patients underwent surgery in the first few weeks.^28^ However, the postoperative neurocognitive complications may cause chronic cognitive dysfunction and higher risk of mortality, which not only happen in the acute phase after surgery.^29^ The occurrence of PND is affected by various factors, including education level, age, gender, etc. Advanced age is an independent risk factor among these factors suggested by clinical studies.^30^ When exposed to systemic stress source such as surgery, many organ systems gradually deteriorate in the process of aging, including the brain.^31^ Consequently, in the present study, we chose aged mice to imitate the clinical condition of PND.

Exploratory laparotomy under the anesthesia of isoflurane is a common approach to induce PND in aged mice,^32^, ^33^ which simulates clinical status closely. Therefore, this approach was chosen to establish the animal model of PND. The surgical trauma caused remarkable neurocognitive dysfunction as indicated by our results of OFT and MWM test. It has been reported that isoflurane alone may induce cognitive dysfunction.^34^, ^35^ The underlying neurotoxicity of isoflurane include elevating ROS production, inducing neuroinflammation,^36^ and disrupting calcium homeostasis.^37^

Microglia is one of the critical facilitators in inducing neuroinflammation, a hallmark of PND. Peripheral inflammation induced by the surgical trauma may activate the microglia staying at a resting condition, contributing to the production of inflammatory cytokines into the CNS and thus leading to neuroinflammation. It has been reported that suppressing the microglia activation inhibited the expression of pro‐inflammatory cytokines, such as TNF‐α and IL‐1β, thereby mitigating neuroinflammation and neurocognitive impairment.^38^ In our study, galectin‐1 inhibited the neuroinflammation in hippocampus, and administered with galectin‐1 not only decreased the peripheral inflammatory cytokines and the protein expression of inflammatory cytokines, but also suppressed microglia activation and disruption of neuronal integrity after surgery in the hippocampus.

It has been verified that the ameliorating role of galectin‐1 in autoimmune inflammation and the inactivation of M1 microglia underlay this kind of neuroprotection, suggesting that the interaction between galectin‐1‐glycan may retard the neurodegeneration.^13^ In the present study, galectin‐1 attenuated the expression of pro‐inflammatory cytokine induced by LPS in BV2 cells. The expression of IL‐1β, IL‐6, TNF‐α, and iNOS is harmfully correlated with the development of neurodegenerative diseases.^39^, ^40^ Suggested by previous studies, using small molecule inhibitors to modulate TNF‐α has the potential for preventing and treating Alzheimer's disease,^41^ and the increased expression level of iNOS in activated microglia contributes to neurodegeneration.^42^ These findings revel that galectin‐1 might exert protective effects on neuroinflammation in neurocognitive disorders.

Suggested by mounting evidence, IRAKs are important mediators in metabolic and inflammatory diseases.^43^, ^44^ IRAK1 deficiency inhibits early pro‐inflammatory cytokine production following polymicrobial sepsis and affects multiple TLR‐dependent pathways,^45^ demonstrating that inhibiting IRAK1 expression may be therapeutic. IRAK1 plays a key role in signal cascade caused by ligand binding to TLRs and IL‐1R.^46^, ^47^ In the present study, we showed that galectin‐1 decreased the protein expression of IRAK1, augmenting the NF‐κB activity, and the expression level of its target genes, including TNFα and IL‐1β.^48^, ^49^

Since IL‐1β and TNF‐α get involved in neuronal survival, their production induced by surgical trauma might lead to neuronal death or morphological abnormalities in the hippocampus, and disrupt cognitive function in aged mice. Thus, the effect of galectin‐1 exerting on the hippocampal neurons following neuroinflammation caused by surgery was explored. As suggested by the results in the study, the number of NeuN‐positive cells significantly reduced after surgery.^50^ Galectin‐1 administration significantly alleviated the disruption of neuronal integrity induced by surgical trauma. This result may account for the phenomenon that galectin‐1 improved memory deficits after surgery. Furthermore, the therapeutic potential of retarding the translocation of c‐Jun and NF‐κB p65 from cytoplasm to nucleus is emphasized in our study, which attenuated the inflammatory response. Collectively, inflammatory mediators, which derived from the activation of AP‐1 and NF‐κB, might mediate in neuronal integrity and neurocognitive dysfunction. The crucial role of the galectin‐1 in surgery‐induced PND demonstrates that treatment blocking the microglia activity, neuronal integrity, and surgery‐mediated inflammatory cytokines could retard the progression of neurocognitive dysfunction.

There are several limitations in our study. First, sexual dimorphism for microglial responses has been reported,^51^ and the present study was done using male mice only. Additionally, Iba‐1 expression is found in both brain microglia and blood‐derived brain infiltrating macrophages.^52^ Therefore, the effect of galectin‐1 exerted on macrophages cannot be ruled out. Finally, although MWM is a robust and reliable test to evaluate hippocampal‐dependent memory, some more behavioral experiments, such as Barnes maze and contextual fear conditioning, are needed to better elucidate the effect of galectin‐1 on cognitive function.

## 5 CONCLUSIONS

In summary, galectin‐1 administration attenuated surgery‐induced neuroinflammation and cognitive dysfunction by decreasing IRAK1 expression. Conversely, overexpressing IRAK1 contributed to increased transcriptional activity and translocation of c‐Jun and NF‐κB p65 in microglia BV2 cells. To sum up, our findings demonstrate that galectin‐1 alleviates inflammatory response and exerts an ameliorating effect in surgery‐induced neurocognitive disorders, which might be a potential therapeutic for decelerating the progression of PND.

## CONFLICT OF INTEREST

The authors declare that they have no competing interests.

## AUTHORS’ CONTRIBUTION

Zhiwen Shen, Hui Xu and Wen Song: involved in investigation, methodology, and writing—Original draft preparation. Chuwen Hu: involved in conceptualization and data curation. Mingyan Guo: involved in visualization, software, and supervision. Jinfeng Li and Junhua Li: involved in writing—reviewing and editing.

## Supporting information

App S1

Tab S1

## Data Availability

All data and models generated or used during the study appear in the submitted article.
