# Supplementary material for: Galectin‐1 ameliorates perioperative neurocognitive disorders in aged mice
Source: CNS Neurosci Ther. 2021 May 4;27(7):842–56. doi: 10.1111/cns.13645 (PMC8193703; doi:10.1111/cns.13645)
Supplement: Supplementary file 1 — App S1 [file CNS-27-842-s002.docx]

**Figure 5D-iNOS-130 kDa**

**
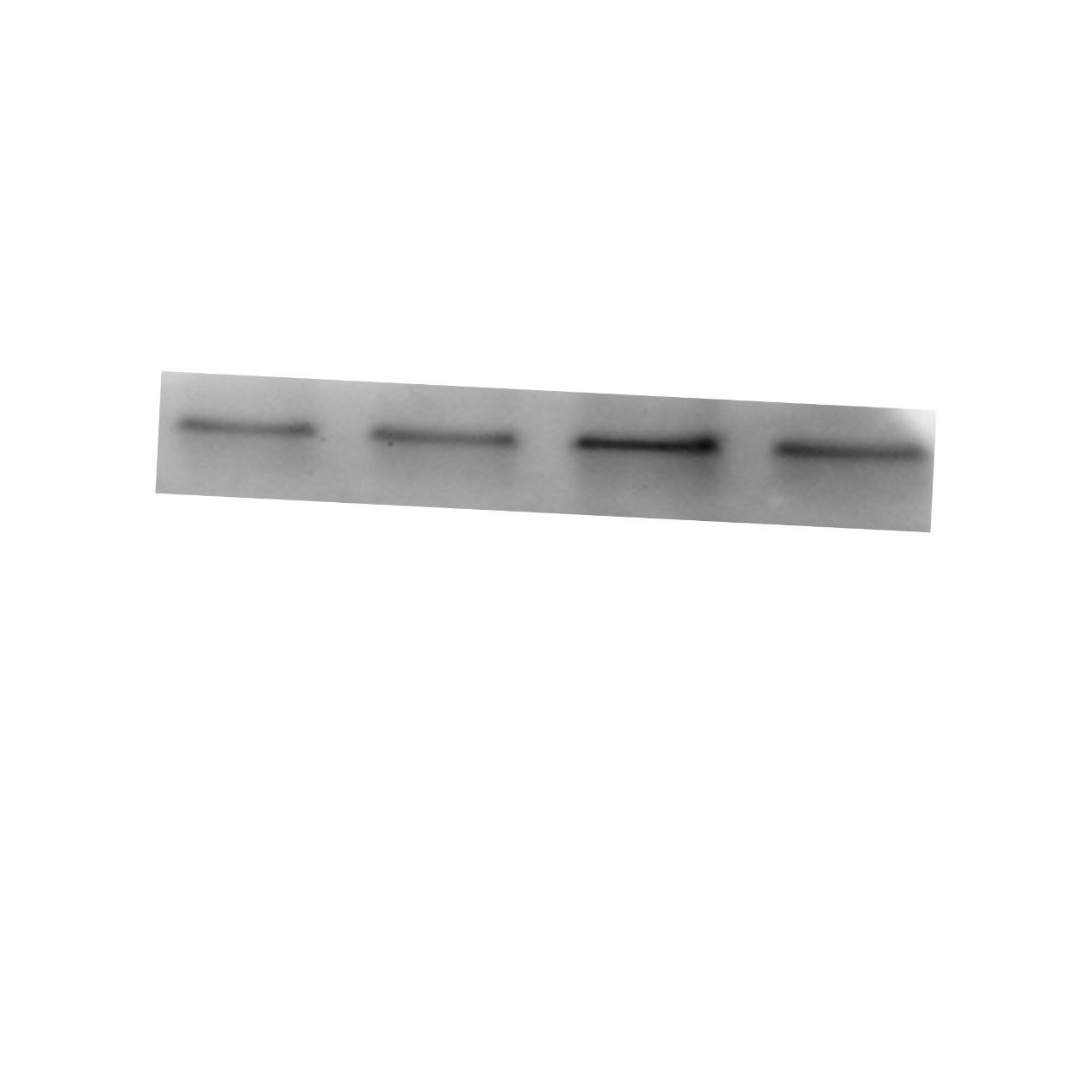
**

**Figure 5D-β-actin-42 kDa**

**
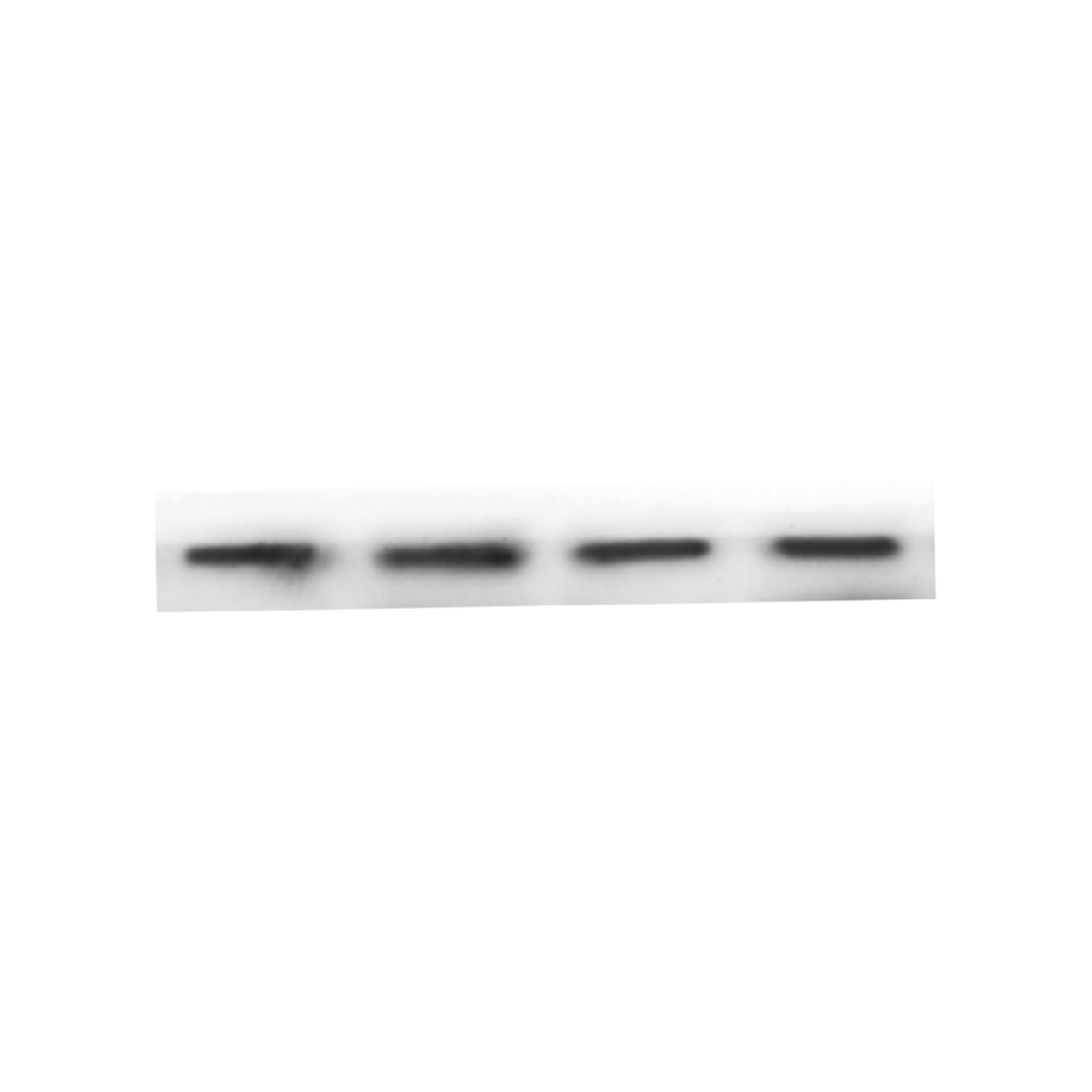
**

**Figure6D-IL-1β-31 kDa**

**
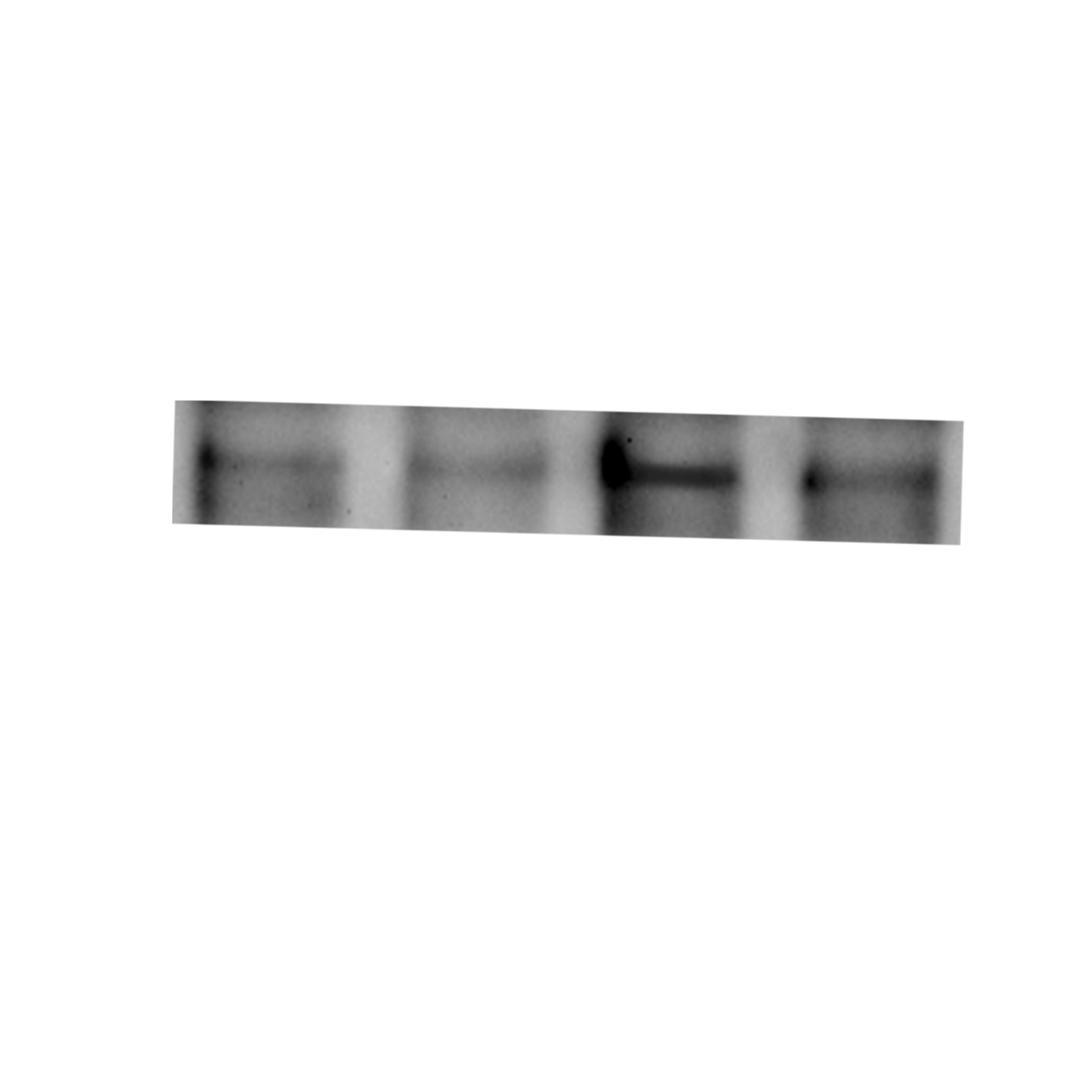
**

**Figure6D-IL-6-24 kDa**

**
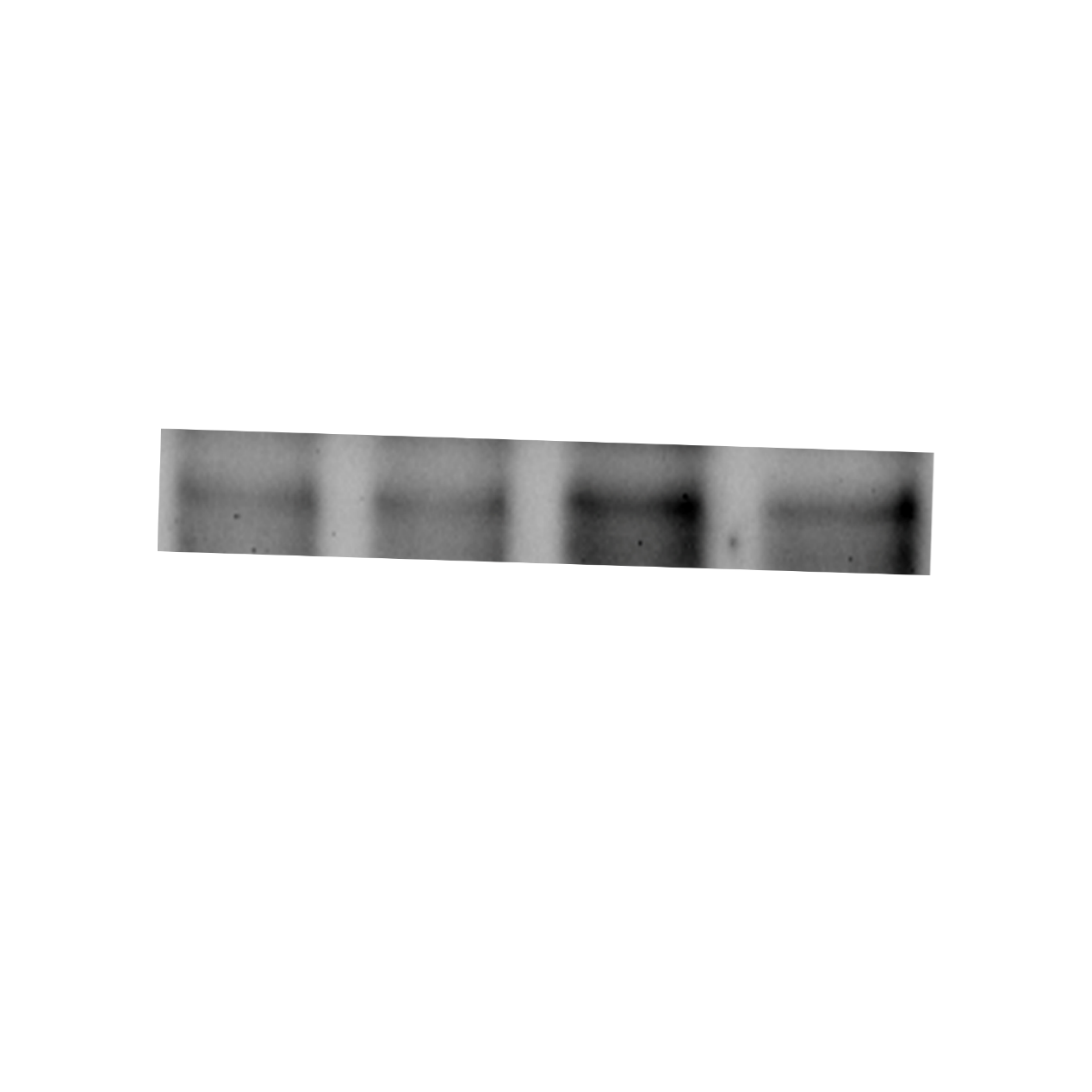
**

**Figure6D-TNF-α-25 kDa**

**
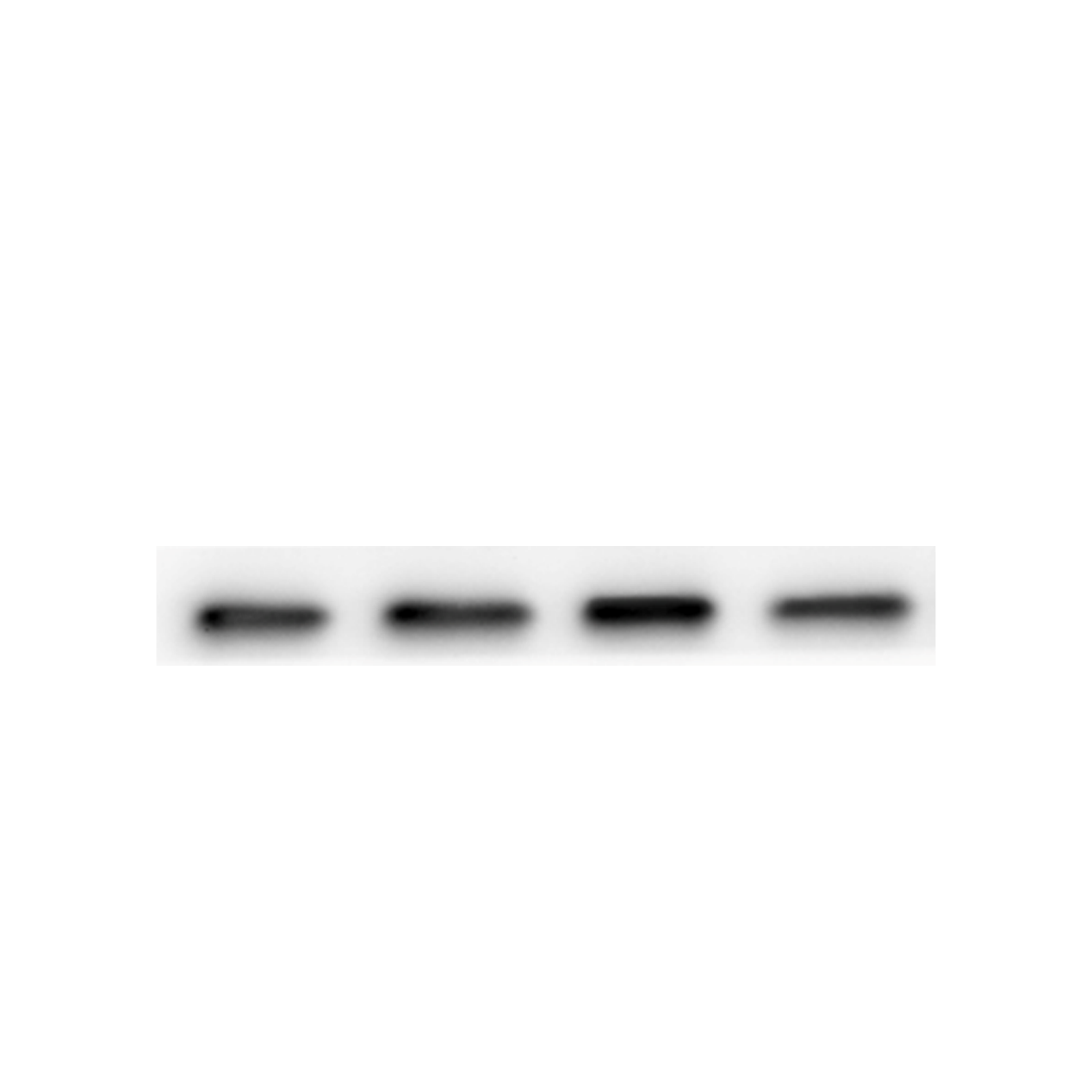
**

**Figure6D-β-actin-42 kDa**

**
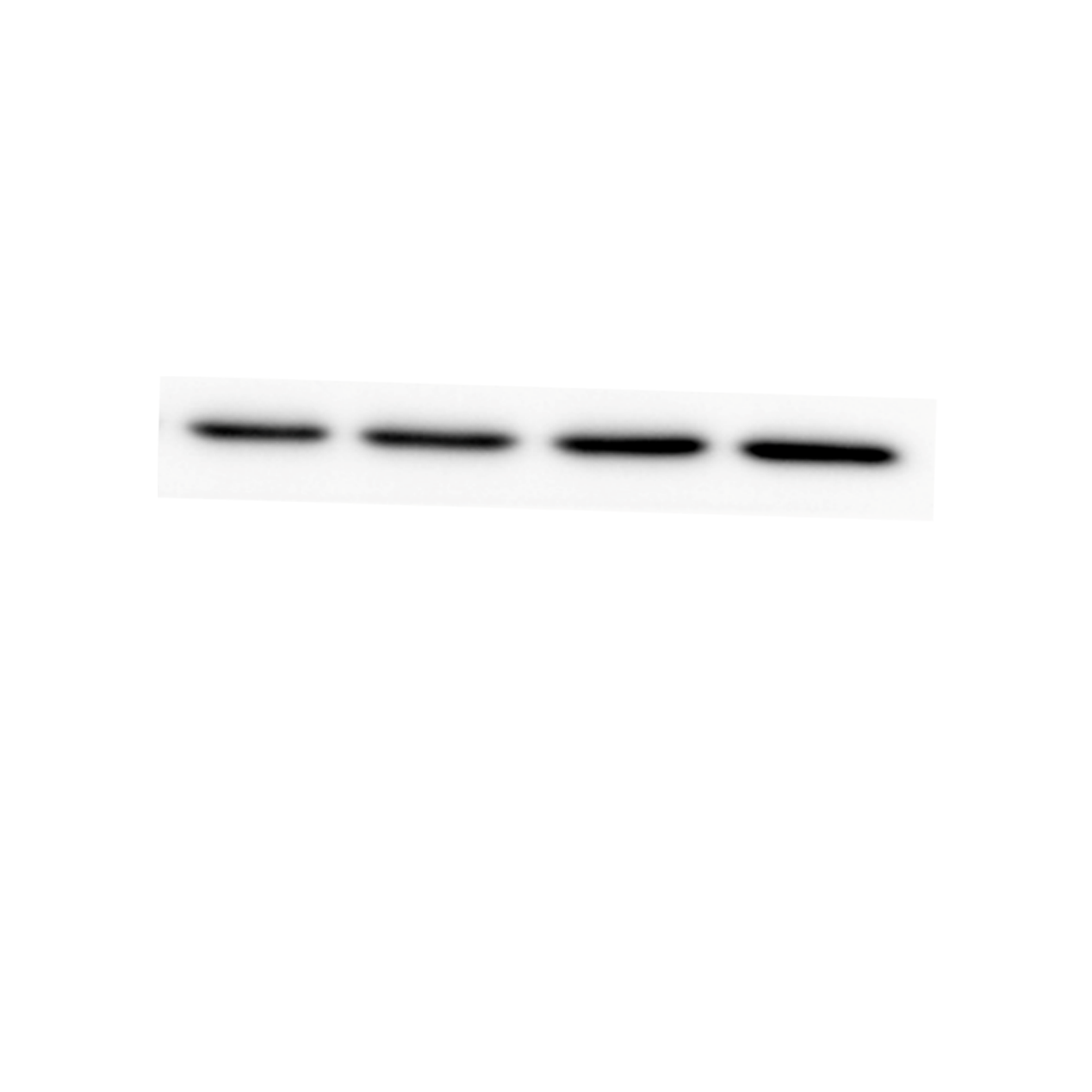
**

**Figure7A-c-Jun-43 kDa**

**
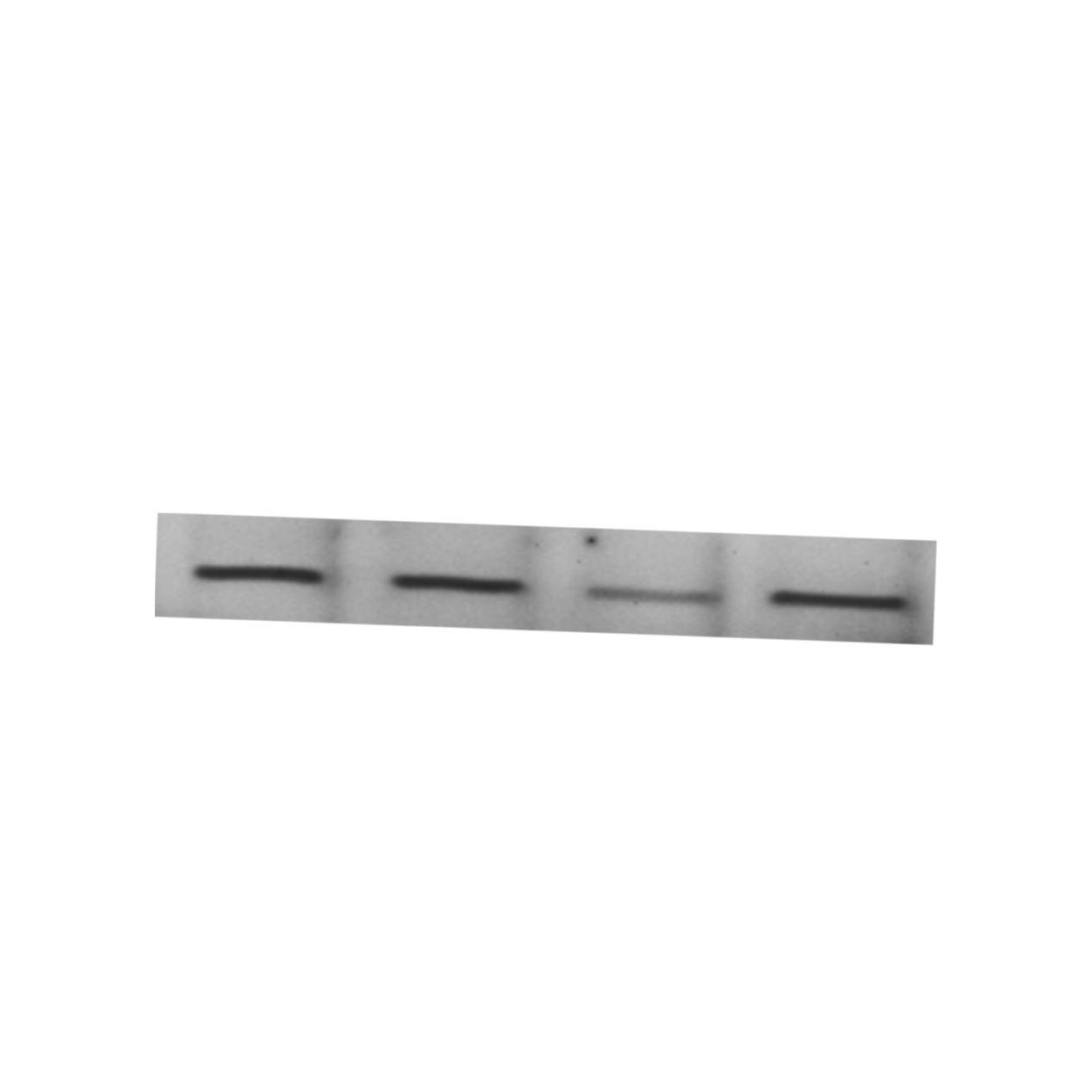
**

**Figure7A-p65-65 kDa**

**
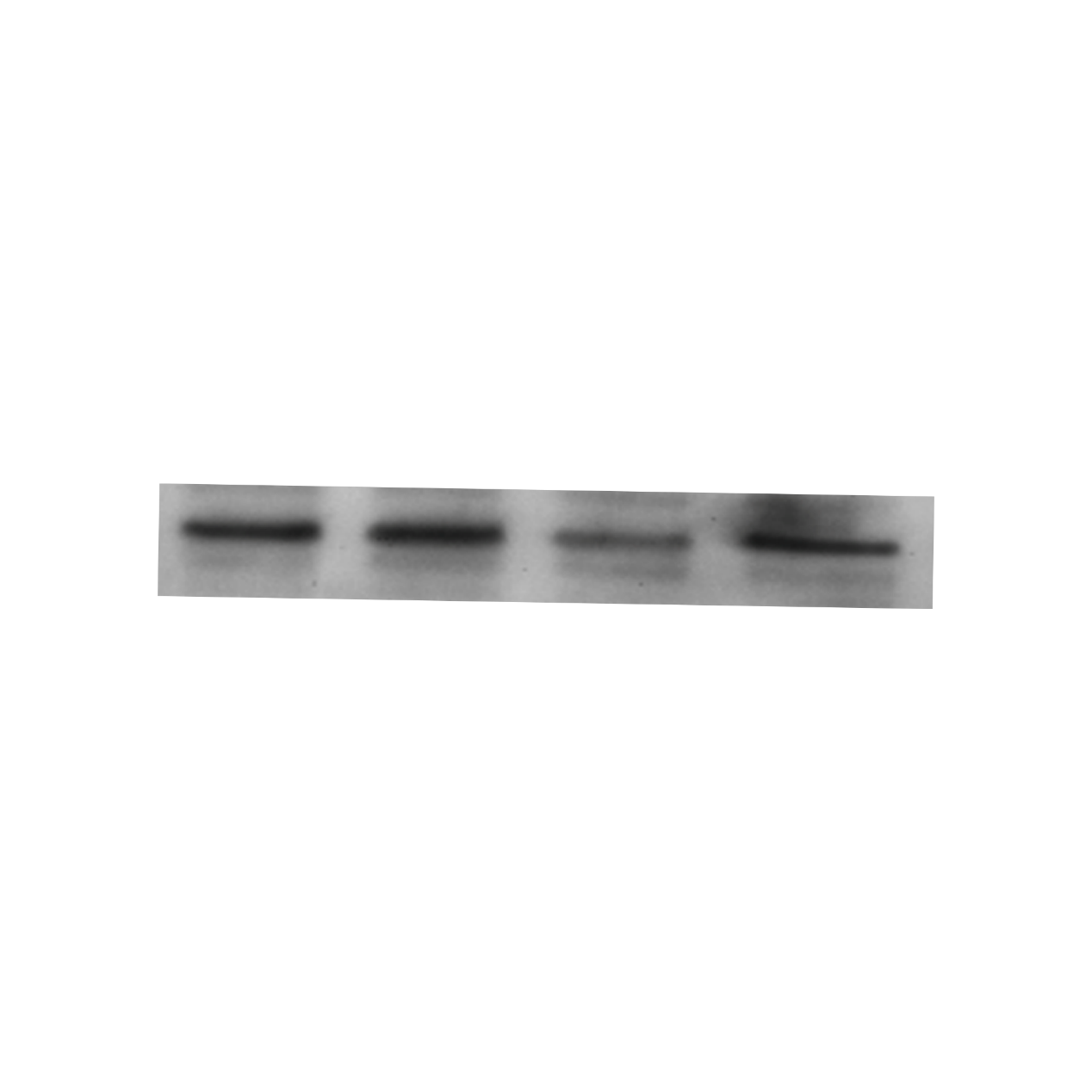
**

**Figure7A-β-actin-42 kDa**

**
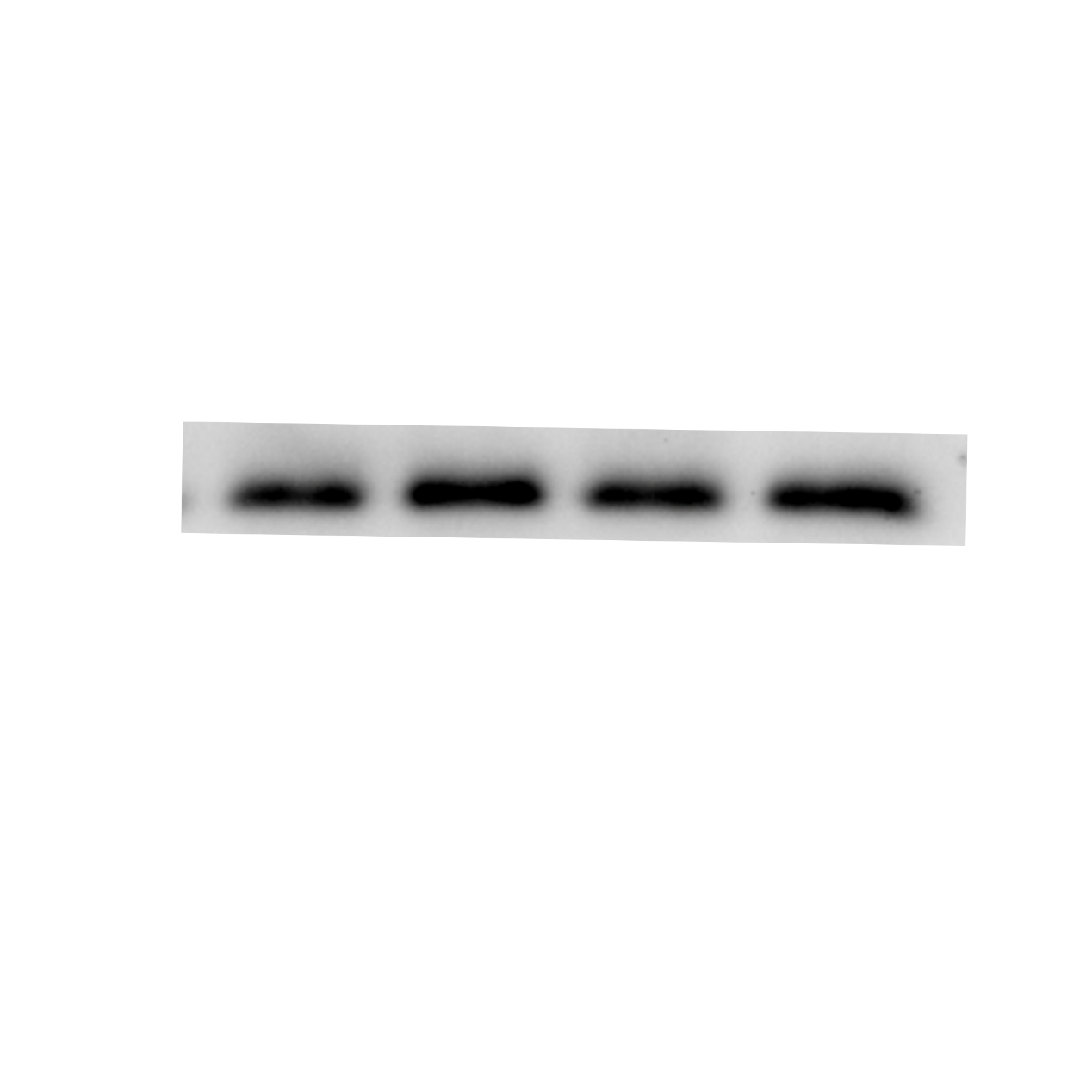
**

**Figure7D-c-Jun-43 kDa**

**
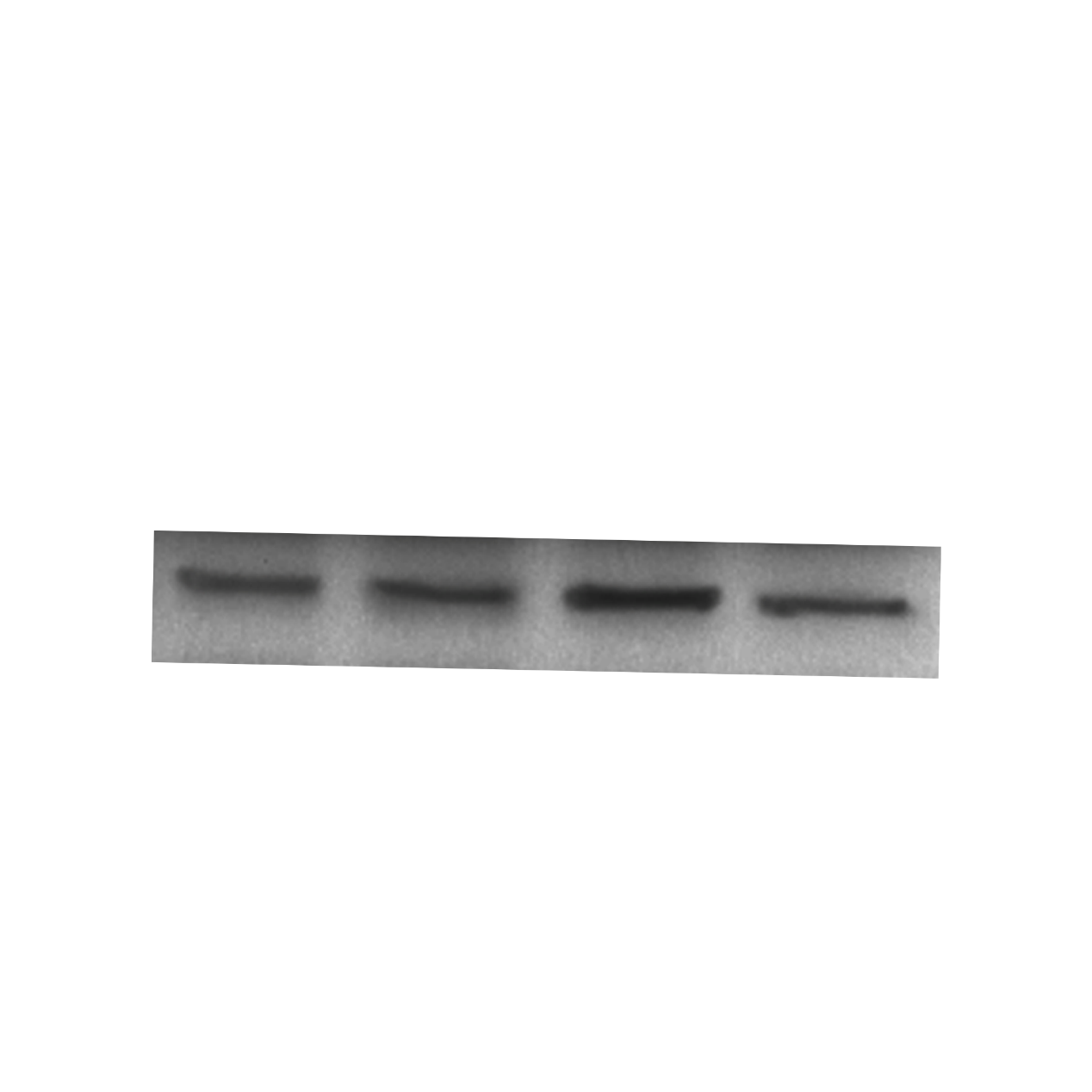
**

**Figure7D-p65-65 kDa**

**
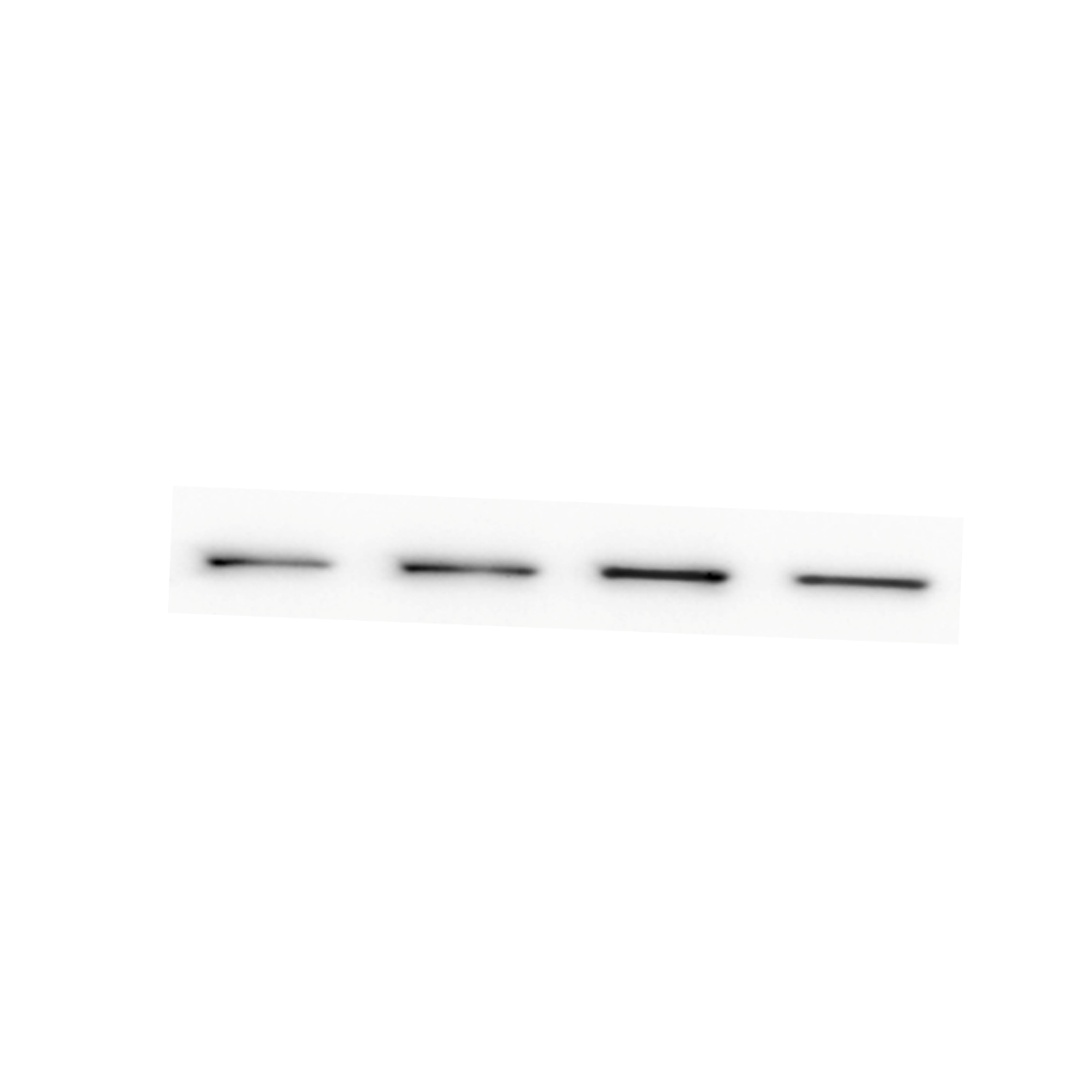
**

**Figure7D-β-actin-42 kDa**

**
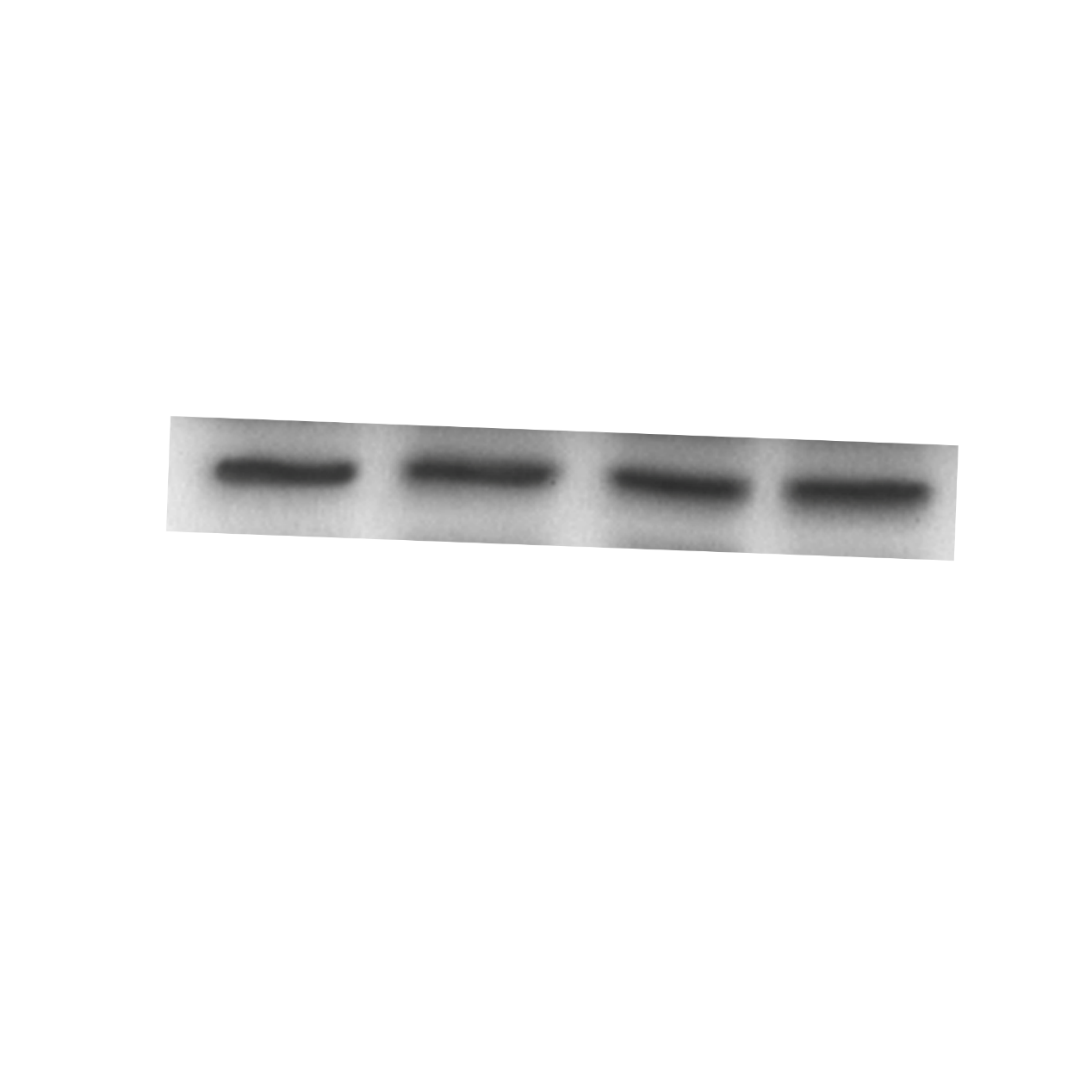
**

**Figure8A-IRAK1-80 kDa**

**
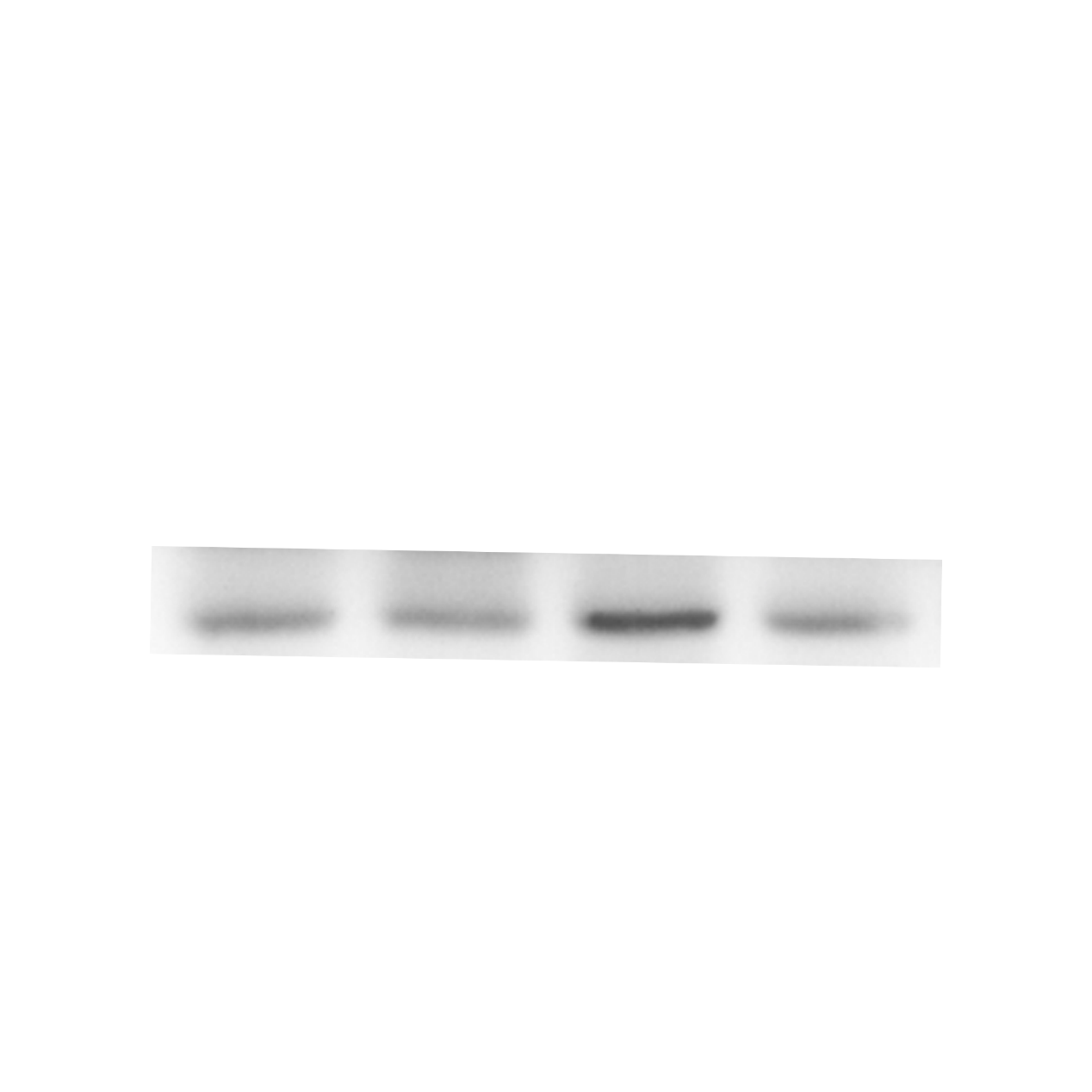
**

**Figure8A-β-actin-42 kDa**

**
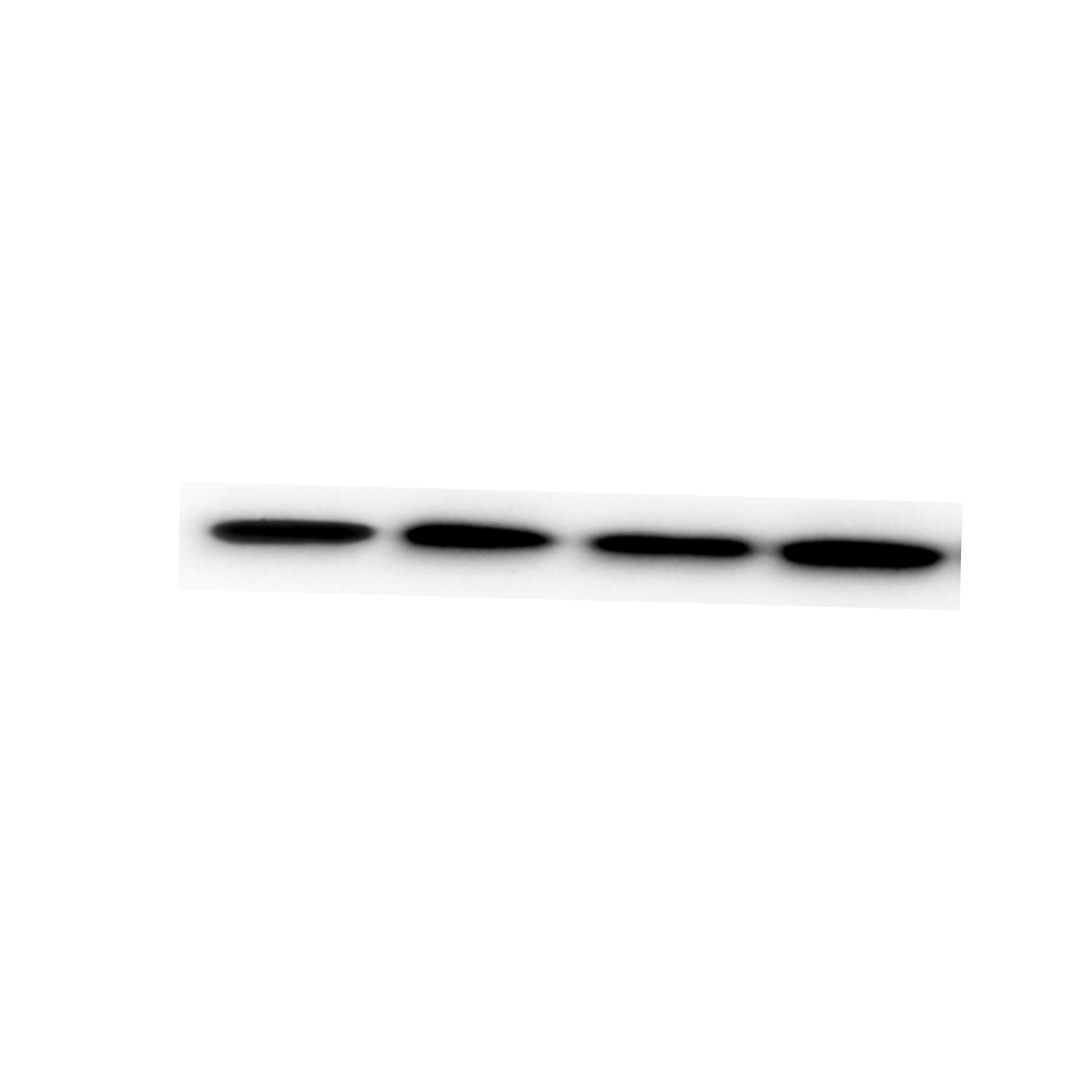
**

**Figure8F-c-Jun-43 kDa**

**
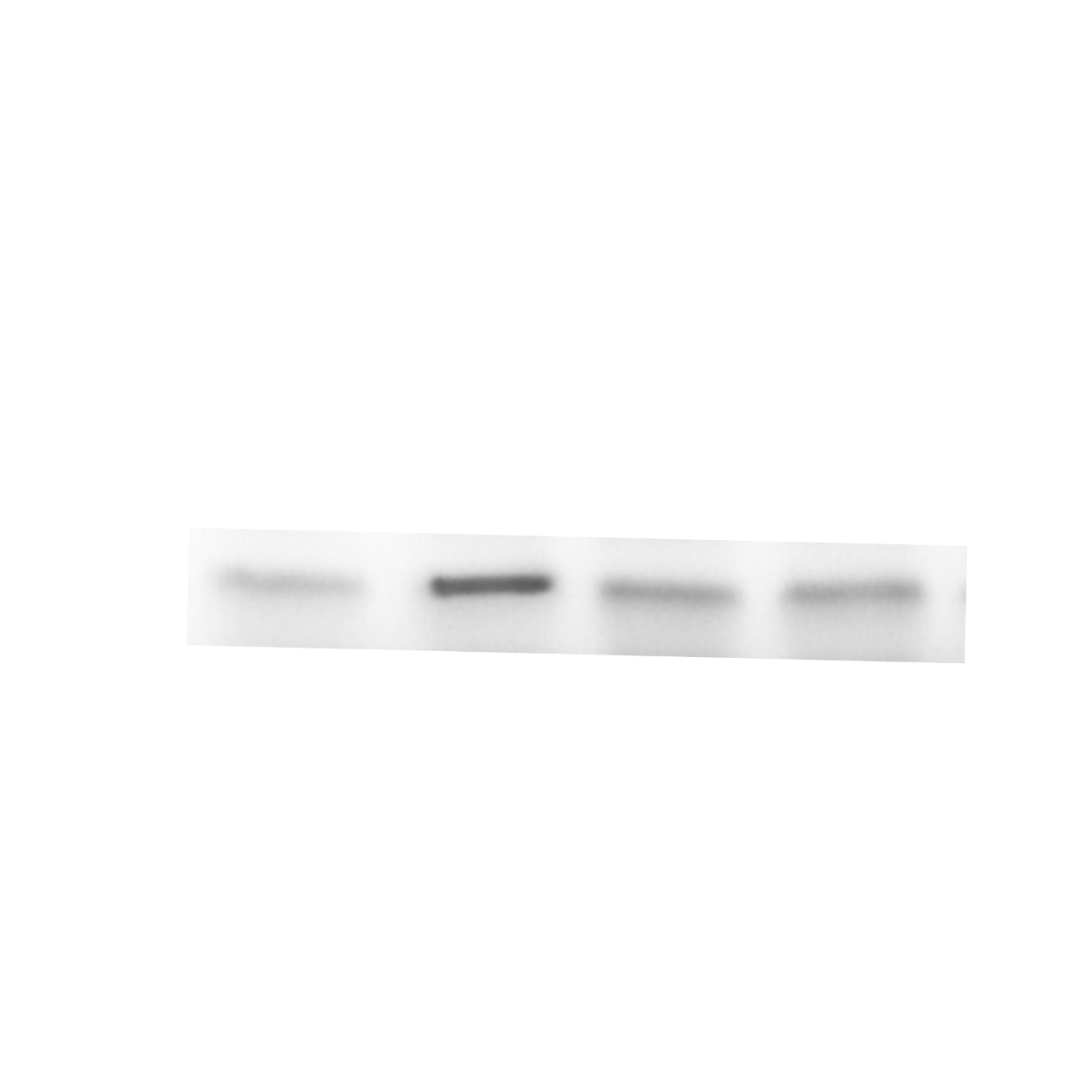
**

**Figure8F-p65-65 kDa**

**
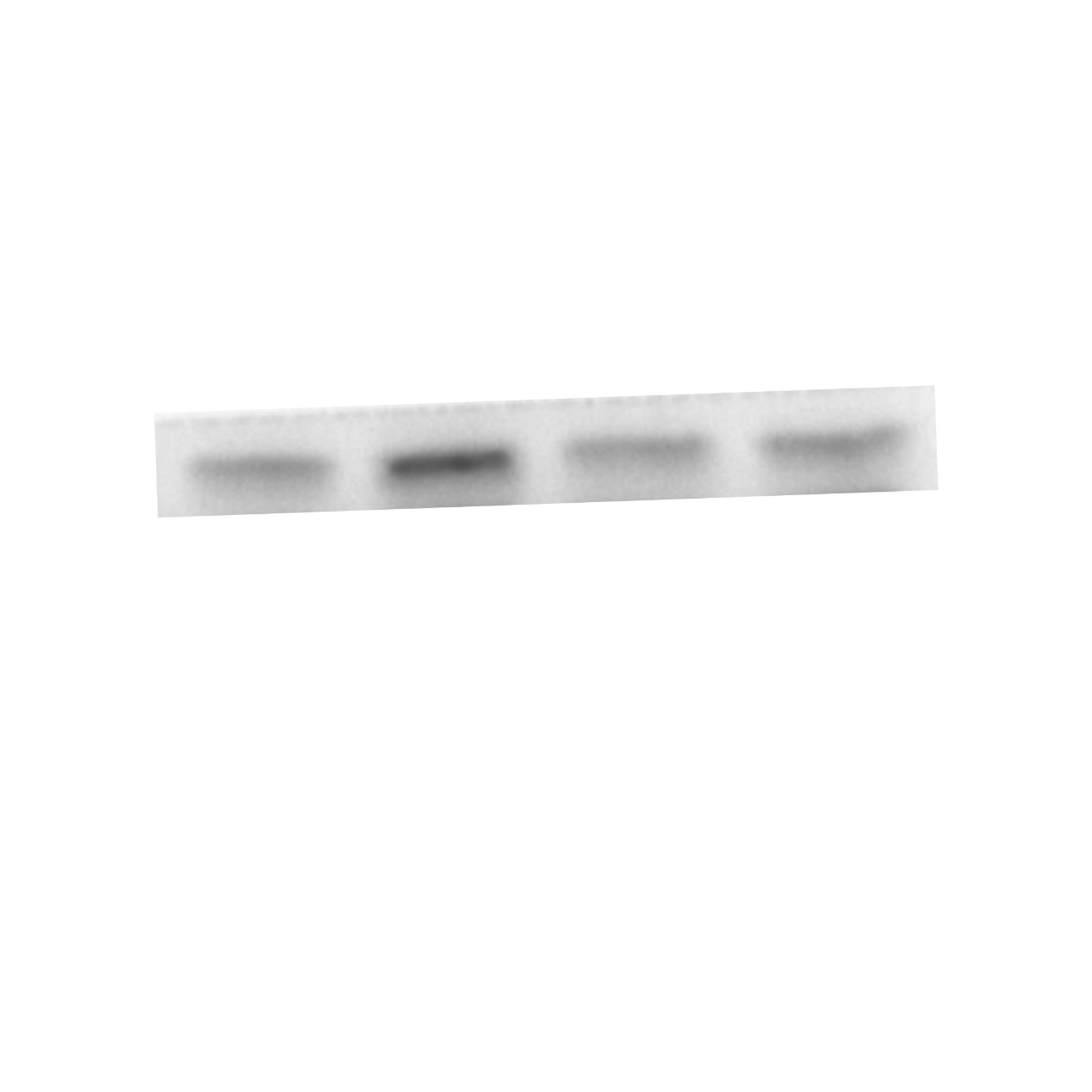
**

**Figure8F-β-actin-42 kDa**

**
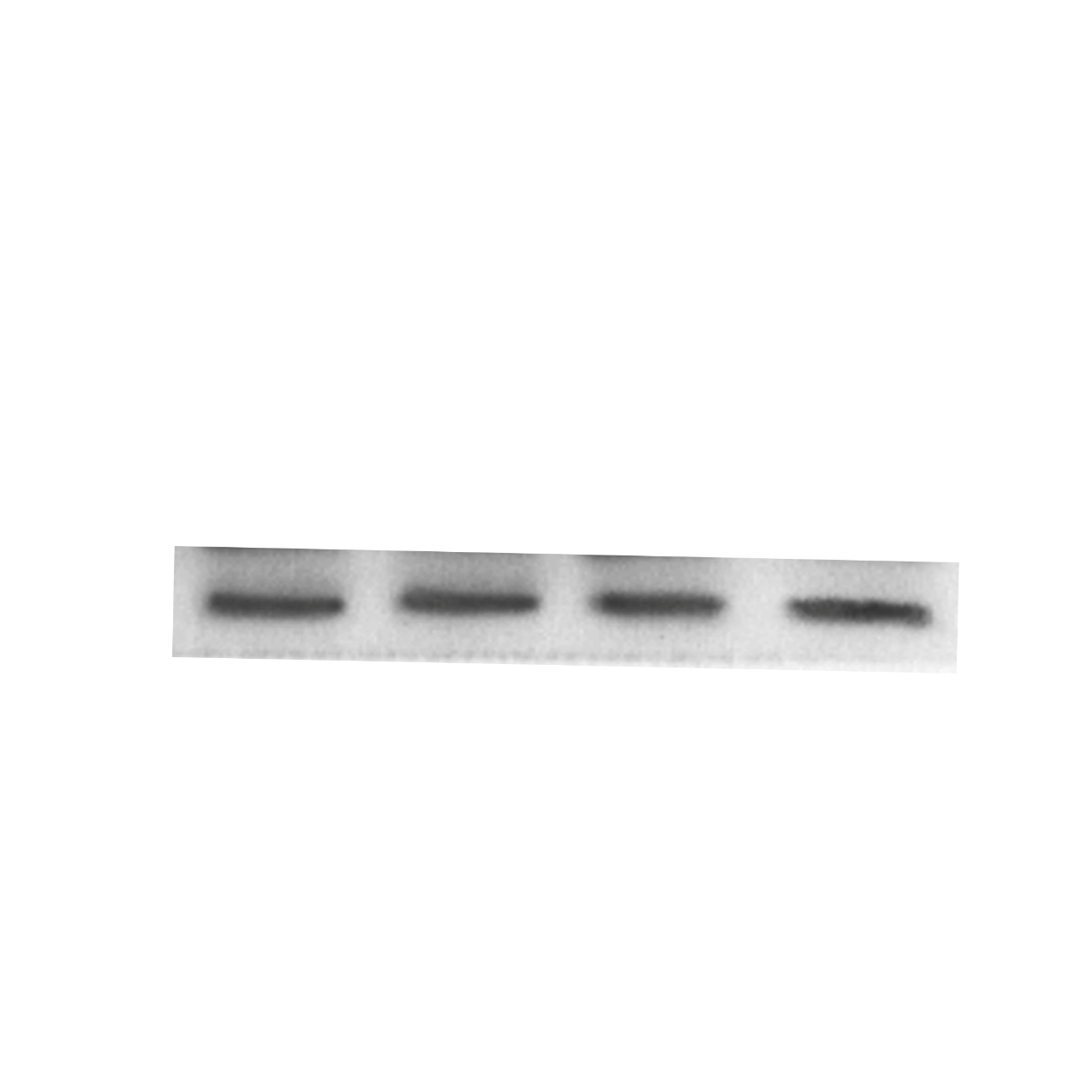
**

**Figure8I-c-Jun-43 kDa**

**
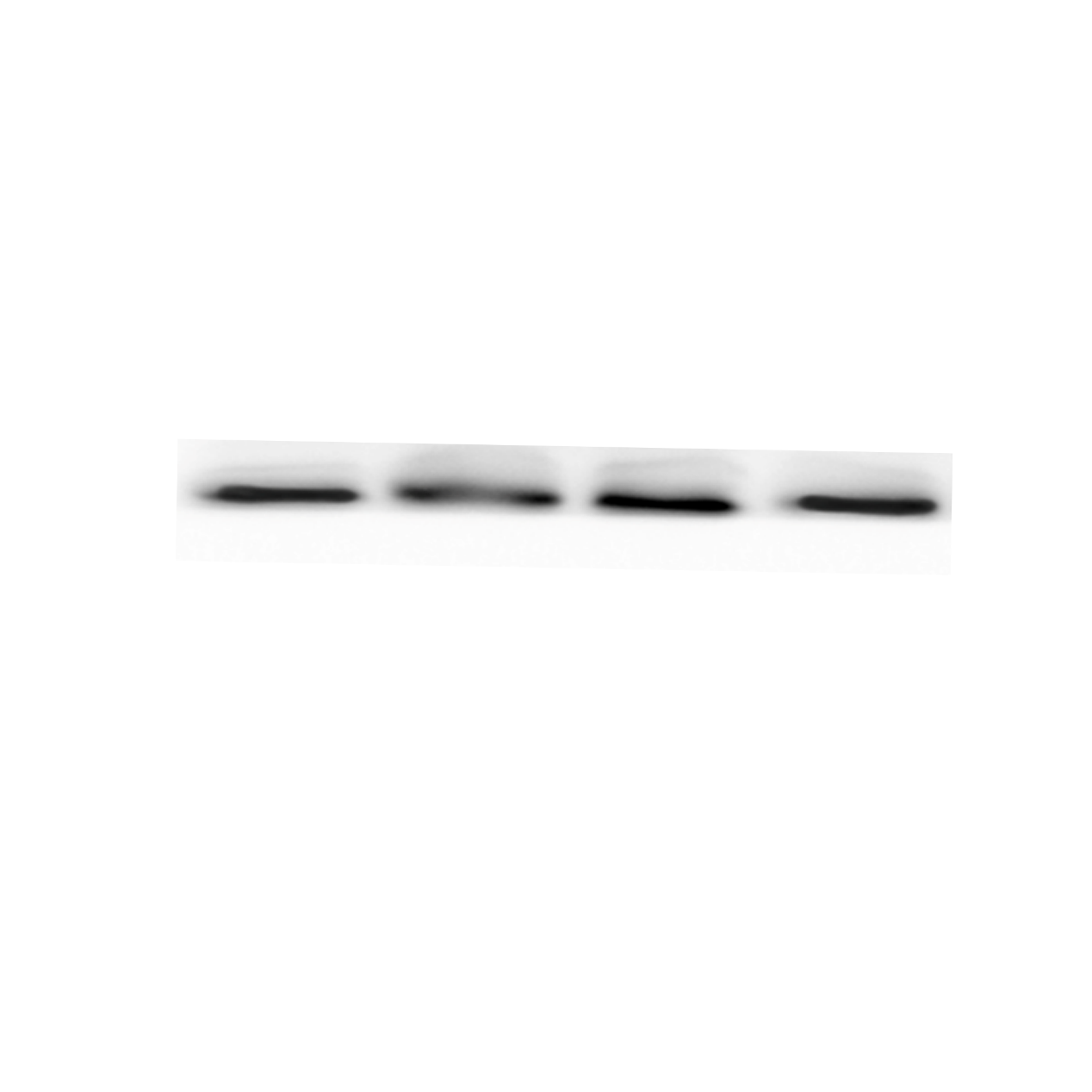
**

**Figure8I-p65-65 kDa**

**
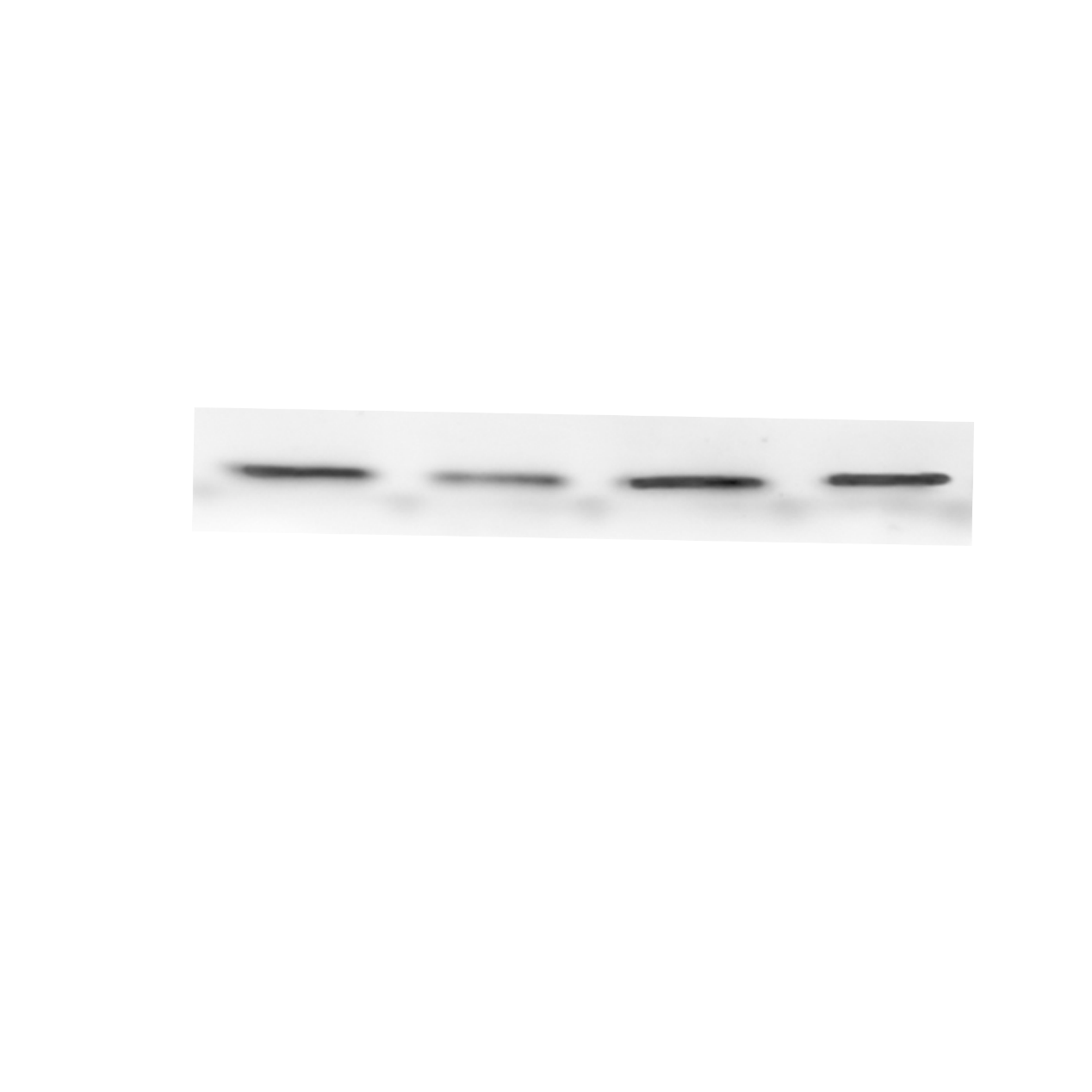
**

**Figure8I-β-actin-42 kDa**

**
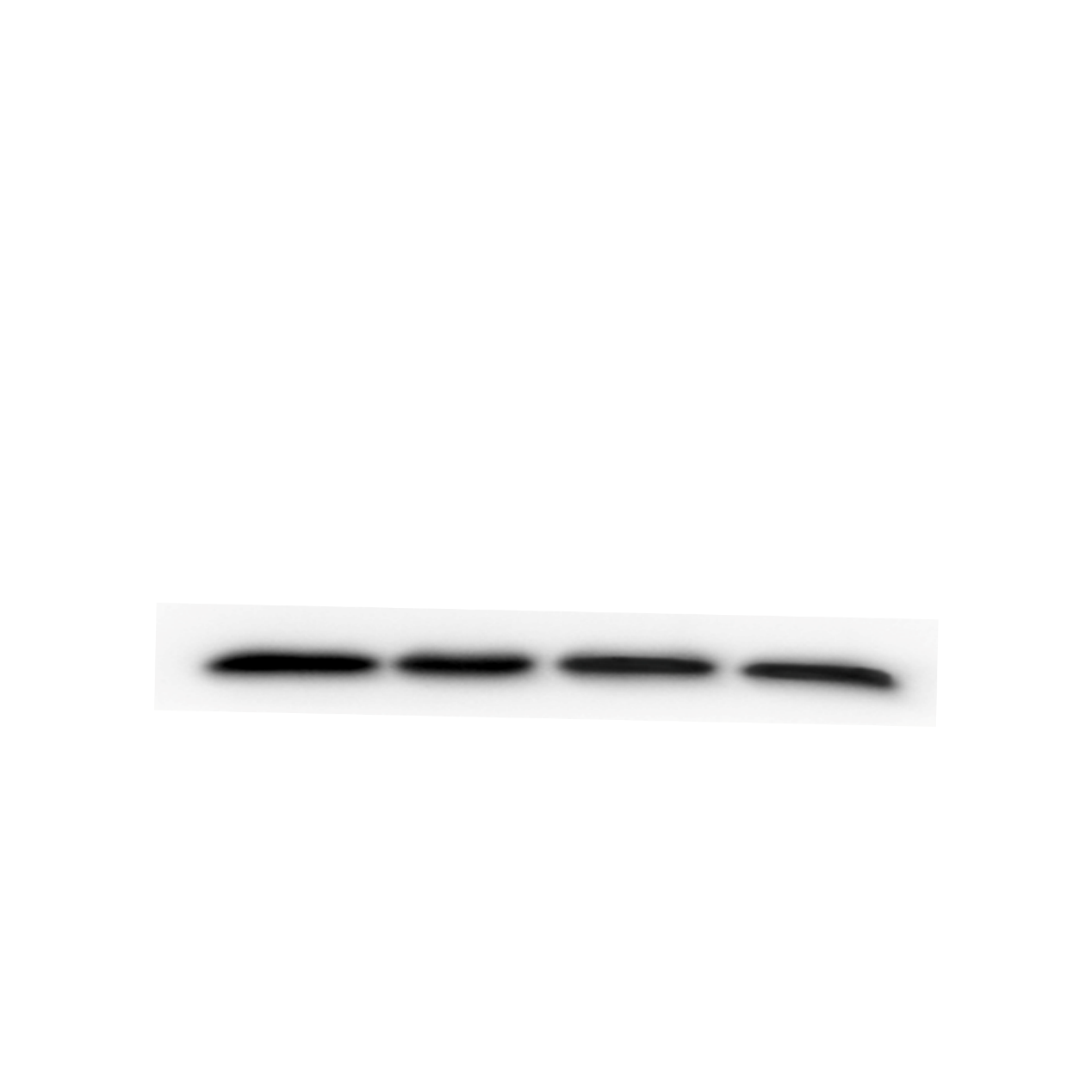
**
