# Supplementary material for: Galectin‐1 ameliorates perioperative neurocognitive disorders in aged mice
Source: CNS Neurosci Ther. 2021 May 4;27(7):842–56. doi: 10.1111/cns.13645 (PMC8193703; doi:10.1111/cns.13645)
Supplement: Supplementary file 2 — Tab S1 [file CNS-27-842-s001.doc]

**Table S1. Primers for qRT-PCR.**

| **Gene Name** | **Direction** | **Primer Sequence (5’ to 3’)** |
| --- | --- | --- |
| iNOS    IL-1β    IL-6    TNF-α    IRAK1    GAPDH | Forward  Reverse  Forward  Reverse  Forward  Reverse  Forward  Reverse  Forward  Reverse  Forward  Reverse | ATGTCCGAAGCAAACATCAC  TAATGTCCAGGAAGTAGGTG  GAAATGCCACCTTTTGACAGTG  TGGATGCTCTCATCAGGACAG  ACTCACCTCTTCAGAACGAATTG  CCATCTTTGGAAGGTTCAGGTTG  CCTCTCTCTAATCAGCCCTCTG  GAGGACCTGGGAGTAGATGAG  GCACCCACAACTTCTCGGAG  CACCGTGTTCCTCATCACCG  GGAGCGAGATCCCTCCAAAAT  GGCTGTTGTCATACTTCTCATGG |
